# Modelling, analysis and optimal control of Zika virus transmission dynamics based on sterile insect technique

**DOI:** 10.1016/j.idm.2025.08.005

**Published:** 2025-08-23

**Authors:** Zongmin Yue, Yingpan Zhang, Xiangrui Ji

**Affiliations:** School of Mathematics and Data Science, Shaanxi University of Science and Technology, Xi'an, China

**Keywords:** Sterile insect technique (SIT), Zika virus, Basic reproduction number, Optimal control, Environmental transmission, Allee effect

## Abstract

The sterile insect technique (SIT) has emerged as a promising tool for suppressing mosquito-borne diseases. This study develops a Zika virus transmission model integrating SIT, emphasizing both mosquito-borne and environmental aquatic transmission pathways. Unlike eradication-focused approaches, the model targets population suppression through sterile male releases, allowing controlled coexistence of sterile and wild mosquitoes. Dynamical analysis reveals critical thresholds: when the sterile insect release rate *b* < *b*_*p*_ and Allee effects are weak (*r* < *r*_*p*_), the system stabilizes at a coexistence equilibrium; exceeding these thresholds drives population collapse. While low wild mosquito densities may theoretically risk extinction, such levels are epidemiologically insufficient to trigger outbreaks, as viral resurgence requires a critical population density. The basic reproduction number *R*_0_ was derived under coexistence conditions, demonstrating that *R*_0_ > 1 ensures viral persistence. Additionally, a multi-objective optimal control framework prioritizes cost minimization over infection reduction, offering resource-efficient strategies. Environmental transmission, a hallmark of Zika virus, accelerates early infection spread but is effectively mitigated by SIT. These results establish actionable thresholds (*b*_*p*_, *r*_*p*_) for balancing mosquito suppression and disease control, while providing theoretical insights applicable to dengue, malaria, and other arboviral diseases.

## 1 Introduction

Zika virus (ZIKV) is a mosquito-borne virus that belongs to the genus Flavivirus in the family Flaviviridae. It was first detected in rhesus monkeys in Uganda in 1947, followed by evidence of human infection and disease in other African countries in the 1950s (Yuan et al., 2021). In 2015–2016, a severe Zika outbreak occurred in Brazil, and the World Health Organization declared the ZIKV outbreak a public health emergency of international concern on 1 February 2016 (World Health Organization, 2022a, World Health Organization, 2022b, World Health Organization, 2022c). In 2020, a total of 22,885 cases of ZIKV were reported in the Americas region. Of these, Brazil reported 18,941 cases, accounting for 83 % of all reported cases in the region (World Health Organization, 2022a, World Health Organization, 2022b, World Health Organization, 2022c). According to the World Health Organization, as of December 2021, 89 countries and territories have reported evidence of mosquito-borne ZIKV infection (World Health Organization, 2022a, World Health Organization, 2022b, World Health Organization, 2022c). ZIKV is transmitted to humans primarily through the bite of infected *Aedes aegypti* mosquitoes and may also be transmitted sexually. Du et al. (2019) showed that aquatic *Aedes aegypti* mosquitoes can be infected with ZIKV from contaminated water environments resulting in adult mosquitoes carrying the virus, and then the infected adult mosquitoes can continue to transmit ZIKV through their bites. This is a new pathway for the transmission of ZIKV, which shortens the transmission cycle of ZIKV considerably, and is likely one of the main reasons for the rapid spread of ZIKV.

To control the spread of ZIKV, the key is to control the mosquito population. Due to the increasing resistance of mosquitoes to insecticides and their ability to adapt to new environments, traditional mosquito control techniques are not sufficient to eliminate mosquitoes. Sterility Insect Technique (SIT) is an effective weapon to control mosquitoes, it is a biological control method that disrupts the natural reproductive process of mosquitoes, using radicals or chemical-physical methods, so that male mosquitoes can not produce offspring. These sterile mosquitoes are then released into the environment to mate with wild mosquitoes present in the environment. Wild females that mate with sterile males do not reproduce, or lay eggs that do not hatch, repeatedly releasing sterile mosquitoes or releasing large numbers of sterile mosquitoes, and ultimately may wipe out or control wild mosquito populations (Alphey et al., 2010). Many scholars have used mathematical models to study the population dynamics between wild mosquitoes and sterile mosquitoes, see (Flores et al., 2003; Diaz et al., 2011; Rafikov et al., 2009; Li, 2014; Dumont et al., 2012; Cai et al., 2014). Li (2017) considered different release strategies for sterile mosquitoes and compared the effects of different release strategies on wild mosquito populations. However, such models do not distinguish between the different growth stages of mosquito populations. The sterile insect technique (SIT) has been successfully implemented in controlling *Aedes aegypti* populations, the primary vector for ZIKA, dengue, and chikungunya viruses. Several studies have explored its potential for Zika containment through field trials and lab-based simulations: Harris et al. (2012) demonstrated that during continuous release of sterile mosquitoes, the wild female mosquito population was reduced by more than 80 %, and the risk of insect-borne virus (e.g., Dengue, ZIKA) transmission significantly declined. Carvalho et al. (2015) demonstrated that sustained release of radiation-sterilized male *Aedes aegypti* in Brazil reduced wild mosquito populations by more than 90 %, indirectly suppressing Zika transmission in pilot areas. While existing studies confirm SIT suppresses Zika transmission, a mathematical model linking sterile mosquito release to viral containment is still lacking.

Mosquitoes undergo four distinct developmental stages throughout their lives: egg, larva, pupa and adult. Interspecific competition between released sterile mosquitoes and wild mosquitoes can lead to increased larval mortality or reduced growth rates. Furthermore, intraspecific competition among wild mosquito larvae may serve as a key driver of density-dependent regulation - specifically, higher larval densities result in greater mortality(Dye., 1982; Gleiser et al., 2000). Crowding essentially occurs in the first three aquatic stages of the mosquito life cycle, i.e., the density dependence of mosquito populations exists mainly in the larval stage. Therefore, it is necessary to introduce mosquito larvae into the modelYakob et al. (2008); White et al. (2010); Lu et al. (2011); Stone et al. (2013). Li. (2009, 2011, 2012); Li et al. (2017) established a stage-structured mosquito population model with sterile mosquitoes, and incorporated factors such as growth and mortality rates between different stages of development in the wild, these models more accurately reflect the growth of mosquito populations. Yin, Yang, Zhang, and Li (2018a, 2018b) in malaria transmission, pointed out that the effect of releasing sterile mosquitoes at a constant rate on the population size of wild mosquitoes, this release strategy is clearly unreasonable, in order to have a more cost-effective release strategy of sterile mosquitoes in the area of the relatively small size of wild mosquitoes, rather than the continuous release of aseptic mosquitoes, and then they optimised the release strategy (Yin et al., 2018a, 2018b), taking into account that the release ratio in sterile mosquitoes is proportional to the population size of wild mosquitoes, proposed a model for malaria transmission with sterile mosquitoes, and ultimately showed that when the release rate is large enough, all wild mosquitoes are eventually eliminated and thus the disease disappears from the population.

Based on the above-mentioned studies, in this paper, we will consider different stages of metamorphosis in wild mosquito populations, and develop a model of ZIKV with stage-structured wild sterile mosquitoes and environmental transmission pathways, and we will analyse the release rate of sterile mosquitoes as well as the effect of contaminated water environment on virus transmission. The full paper is organised as follows: In Section 2, a new ZIKV model is proposed and the boundedness of the model solution is investigated. In Sections 3, 4, the model is analysed qualitatively, and the main results on stability and expressions for the basic regeneration number are obtained. The consistency persistence of the model is also discussed. In Section 5, based on the practical situation, the control strategy is proposed, the optimal control model is established and the optimal control analysis is carried out. In Section 6, numerical simulations are given based on the developed mathematical model. The optimal control strategies with different control cost weights are compared and analysed. Section 7 gives the conclusion.

## 2 A model of ZIKV transmission

Li et al. (2017) presents a stage-structured mosquito population model with sterile mosquitoes:

(1) $$\left\{\begin{array}{ll} & \frac{dJ}{dt}=\beta \left(\cdot \right)N_{V}-\alpha \left(1-\kappa \left(J\right)\right)-\left(d_{0}+d_{1}J\right)J,\\ & \frac{dN_{V}}{dt}=\alpha \left(1-\kappa \left(J\right)\right)-\mu_{1}N_{V},\\ & \frac{dG}{dt}=B\left(N_{V}\right)-\mu_{2}G. \end{array}\right)$$

Let *G*(*t*) be the population of sterile mosquitoes at time *t*, since these mosquitoes do not give birth, the birth rate of sterile mosquitoes is the release ratio in mosquitoes *B*(*N*_*V*_). Here, *β*(⋅) is the birth rate of adult wild mosquitoes (i.e., the oviposition rate), the plumage rate of wild mosquitoes from larvae to adults is a function of larvae, in the form of *α*(1 − *κ*(*J*)), *α* is the maximum rate of plumage, *κ*(*J*) is a functional response due to intraspecific competition, 0 ≤ *κ*(*J*) ≤ 1, *κ*(0) = 0, *κ*′(*J*) > 0 and $\underset{J\to \infty }{\lim}\kappa \left(J\right)=1$. Assuming that density dependence exists only in the larval stage, *d*_0_, *d*_1_ denote the natural mortality rate of wild mosquito larvae and the mortality rate due to intraspecific competition, respectively, and *μ*_1_, *μ*_2_ are the mortality rate of adult wild mosquitoes and the mortality rate of sterile mosquitoes, respectively.

Following (Yin et al., 2018a, 2018b), we assume that the release rate of sterile mosquitoes, *B*(*N*_*V*_), is proportional to the population size of wild mosquitoes, formulated as *B*(*N*_*V*_) = *bN*_*V*_, where *b* represents the proportionality constant. This strategy suppresses the proliferation of wild mosquitoes by interfering with their mating processes. Significantly, the intervention induces an Allee effect (Allee, 1931; Dennis., 1989; Yue & Wang, 2022), wherein low population densities result in reduced mating efficiency due to limited mate-finding opportunities. Specifically, individuals within a population exhibit diminished survival or reproductive success under extremely small population sizes. In the context of our wild mosquito study, the Allee effect manifests as adult mosquitoes encountering difficulties in locating mates at low population densities, thereby causing a decline in reproduction rates. To quantify this phenomenon, we formalize the relationship as(Yue & Wang, 2022; Li et al., 2017):

$$\beta \left(\cdot \right)=\frac{\sigma N_{V}}{N_{V}+G+\gamma }N_{V},$$

where *γ* is the Allee effect constant, and *σ* represents the number of offspring produced by a wild mosquito per unit of time through all matings.

This study posits that mosquitoes attain adulthood at the highest emergence rate, denoted as *α*, where *κ*(*J*) = 0. Intraspecific competition primarily results in increased mortality, denoted as *d*_1_. Consequently, for mosquito larvae, the following applies:

$$\frac{dJ}{dt}=\frac{\sigma N_{V}}{N_{V}+G+\gamma }N_{V}-\alpha J-\left(d_{0}+d_{1}J\right)J.$$

The environmental transmission route of the Zika virus involves wild mosquitoes acquiring the virus during the larval stage from their surroundings. Hence, we categorize the larvae J into two groups: susceptible larvae (*S*_*J*_) and infected larvae (*I*_*J*_). The adult mosquito population (*N*_*V*_) is also divided into two classes: susceptible (*S*_*V*_) and infected (*I*_*V*_) individuals. To couple with human infection pathways, the human population is categorized into three compartments: susceptible (*S*_*H*_), infected (*I*_*H*_), and recovered (*R*_*H*_) individuals, with the total population satisfying *N*_*H*_ = *S*_*H*_ + *I*_*H*_ + *R*_*H*_. We define *V*(*t*) as the Zika virus (ZIKV) concentration in contaminated aquatic environments. The synthesis of these components yields the following Zika virus transmission model:

(2) $$\left\{\begin{array}{ll} & \frac{dS_{H}}{dt}=\Lambda -r\beta_{H}I_{V}S_{H}-\mu_{H}S_{H},\\ & \frac{dI_{H}}{dt}=r\beta_{H}I_{V}S_{H}-\left(\mu_{H}+\delta +\eta \right)I_{H},\\ & \frac{dR_{H}}{dt}=\eta I_{H}-\mu_{H}R_{H},\\ & \frac{dS_{J}}{dt}=\frac{\sigma N_{V}}{N_{V}+G+\gamma }N_{V}-\alpha S_{J}-\beta S_{J}V-\left(d_{0}+d_{1}\left(S_{J}+I_{J}\right)\right)S_{J},\\ & \frac{dI_{J}}{dt}=\beta S_{J}V-\alpha I_{J}-\left(d_{0}+d_{1}\left(S_{J}+I_{J}\right)\right)I_{J},\\ & \frac{dS_{V}}{dt}=\alpha S_{J}-r\beta_{V}I_{H}S_{V}-\mu_{1}S_{V},\\ & \frac{dI_{V}}{dt}=\alpha I_{J}+r\beta_{V}I_{H}S_{V}-\mu_{1}I_{V},\\ & \frac{dV}{dt}=\theta I_{H}-\mu V,\\ & \frac{dG}{dt}=bN_{V}-\mu_{2}G, \end{array}\right)$$

All parameters are positive, as listed in Table 1. Considering the biological significance of the model, *σ* − *α* − *d*_0_ > 0. In the ZIKV transmission model, we primarily concentrate on the mosquito-borne transmission pathway while excluding human-to-human transmission. This approach is justified because mosquito-borne transmission represents the predominant route for ZIKV spread, demonstrating significantly higher transmission efficiency and broader geographical impact compared to other potential modes(World Health Organization, 2022a, World Health Organization, 2022b, World Health Organization, 2022c; Gregory et al., 2017). Furthermore, focusing on mosquito-borne transmission not only corresponds to the virus's epidemiological profile but also reduces the model's complexity. This streamlined approach enables effective evaluation of mosquito control strategies, particularly the SIT implementation that constitutes the central focus of this investigation.

Lemma 1 *The solutions* (*S*_*H*_, *I*_*H*_, *R*_*H*_, *S*_*J*_, *I*_*J*_, *S*_*V*_, *I*_*V*_, *V*, *G*) *of model* (2) *are non-binding for all* *t* > 0.

***Proof*.** *The dynamical equation for the total population is*

$$\frac{dN_{H}}{dt}=\Lambda -\mu_{H}N_{H}-\delta I_{H}\leq \Lambda -\mu_{H}N_{H},$$

*then, when* *t* → *∞,* $0\leq N_{H}\leq \frac{\Lambda }{\mu_{H}}$*.*

*The dynamical equation for wild mosquito larvae is*

$$\frac{dJ}{dt}=\frac{\sigma N_{V}}{N_{V}+G+\gamma }N_{V}-\alpha J-\left(d_{0}+d_{1}J\right)J\leq \left(\sigma -\alpha -d_{0}\right)J-d_{1}J^{2},$$

*when* *t* → *∞,* $0\leq J\leq \frac{\sigma -\alpha -d_{0}}{d_{1}}$*.*

*The dynamical equation for the adult wild mosquito is*

$$\frac{dN_{V}}{dt}=\alpha J-\mu_{1}N_{V}\leq \frac{\alpha \left(\sigma -\alpha -d_{0}\right)}{d_{1}}-\mu_{1}N_{V},$$

*when* *t* → *∞,* $0\leq N_{V}\leq \frac{\alpha \left(\sigma -\alpha -d_{0}\right)}{\mu_{1}d_{1}}$*.*

*For sterile mosquitoes*, *we have*

$$\frac{dG}{dt}=bN_{V}-\mu_{2}G\leq \frac{\alpha b\left(\sigma -\alpha -d_{0}\right)}{\mu_{1}d_{1}}-\mu_{2}G,$$

*then when* *t* → *∞,* $0\leq G\leq \frac{\alpha \left(\sigma -\alpha -d_{0}\right)}{\mu_{1}\mu_{2}d_{1}}$*.*

*For the concentration of ZIKV in contaminated aquatic environments*, *we get*

$$\frac{dV}{dt}=\theta I_{H}-\mu V\leq \frac{\theta \Lambda }{\mu_{H}}-\mu V,$$

*when* *t* → *∞,* $0\leq V\leq \frac{\theta \Lambda }{\mu \mu_{H}}$*.*

*Thus*

$$\begin{array}{c} \underset{t\to \infty }{\lim}\sup N_{H}\left(t\right)\leq \frac{\Lambda }{\mu_{H}},\underset{t\to \infty }{\lim}\sup\;J\left(t\right)\leq \frac{\sigma -\alpha -d_{0}}{d_{1}},\underset{t\to \infty }{\lim}\sup N_{V}\left(t\right)\leq \frac{\alpha \left(\sigma -\alpha -d_{0}\right)}{\mu_{1}d_{1}},\\ \underset{t\to \infty }{\lim}\sup\;G\left(t\right)\leq \frac{\alpha \left(\sigma -\alpha -d_{0}\right)}{\mu_{1}\mu_{2}d_{1}},\underset{t\to \infty }{\lim}\sup\;V\left(t\right)\leq \frac{\theta \Lambda }{\mu \mu_{H}}. \end{array}$$

*Let* *P*(*t*) = *N*_*H*_(*t*) + *J*(*t*) + *N*_*V*_(*t*) + *G*(*t*) + *V*(*t*)*, with derivatives for* *t* *on both sides of the equation, we get:*

$$\frac{dP\left(t\right)}{dt}\leq \Lambda +\frac{{\left(\sigma -\alpha -d_{0}\right)}^{2}}{4d_{1}}+\frac{\alpha \left(\sigma -\alpha -d_{0}\right)}{d_{1}}+\frac{\alpha b\left(\sigma -\alpha -d_{0}\right)}{\mu_{1}d_{1}}+\frac{\theta \Lambda }{\mu_{H}}≜M.$$

*By the principle of differential inequality, for all* *t* ≥ *T* ≥ 0*, there is*

$$0\leq P\left(t\right)\leq M-\left(M-P\left(T\right)\right)e^{-\left(t-T\right)}.$$

Consequently, $\underset{t\to \infty }{\lim}\sup\left(N_{H}+J+N_{V}+G+V\right)\leq M$.

$$\begin{array}{c} \Omega =\left\{\left(S_{H},I_{H},R_{H},S_{J},I_{J},S_{V},I_{V},G,V\right)\in R_{+}^{9}|0\leq S_{H}+I_{H}+R_{H}\leq \frac{\Lambda }{\mu_{H}},0\leq S_{J}+I_{J}\right)\\ \left(\leq \frac{\sigma -\alpha -d_{0}}{d_{1}},0\leq S_{V}+I_{V}\leq \frac{\alpha \left(\sigma -\alpha -d_{0}\right)}{\mu_{1}d_{1}},0\leq G\leq \frac{\alpha b\left(\sigma -\alpha -d_{0}\right)}{\mu_{1}\mu_{2}d_{1}},0\leq V\leq \frac{\theta \Lambda }{\mu \mu_{H}}\right\}. \end{array}$$

*By* Lemma 1, Ω *is a positive invariant set for which model (2) has non-negative initial conditions.*

**Table 1:** Description of the parameters used in model (2).

symbols	implications
Λ	The recruitment rate of the susceptible population
*μ*_*H*_	The natural mortality rate of humans
*δ*	Mortality due to disease
*η*	Recovery rate of infected persons by drugs or natural immunity
*r*	Number of human bites per mosquito per unit of time
*β*_*H*_	Probability of virus transmission from biting susceptible persons by infected mosquitoes
*β*_*V*_	Probability of virus transmission from infected persons bitten by susceptible mosquitoes
*α*	Transformation rates of wild mosquito larvae into adults
*d*_0_	Natural mortality of wild mosquito larvae
*d*_1_	Mortality of wild mosquito larvae owing to intraspecific competition
*μ*_1_	Mortality of adult wild mosquitoes
*μ*_2_	Mortality of sterile mosquitoes
*b*	Release ratio in sterile mosquitoes
*σ*	Number of offspring per wild mosquito per unit of time through all matings
*β*	Probability of virus transmission from contaminated aquatic environments to wild mosquito larvae
*θ*	Defecation rate per infected agent (rate of increase in viral concentration with infected agent)
*μ*	ZIKV clearance in contaminated aquatic environments
*γ*	The Allee effect constant

## 3 Dynamics of mosquito populations

The population dynamics between wild and sterile mosquitoes were modelled without incorporating disease transmission dynamics, as described by the following system of equations:

(3) $$\left\{\begin{array}{ll} & \frac{dJ}{dt}=\frac{\sigma N_{V}}{N_{V}+G+\gamma }N_{V}-\alpha J-\left(d_{0}+d_{1}J\right)J,\\ & \frac{dN_{V}}{dt}=\alpha J-\mu_{1}N_{V},\\ & \frac{dG}{dt}=bN_{V}-\mu_{2}G. \end{array}\right)$$

It is easy to obtain that there exists a zero equilibrium point ${\bar{E}}_{0}=\left(0,0,0\right)$ for model (3). The positive equilibrium point of model (3) satisfies

$$\left\{\begin{array}{ll} & \frac{\sigma N_{V}}{N_{V}+G+\gamma }N_{V}-\alpha J-\left(d_{0}+d_{1}J\right)J=0,\\ & \alpha J-\mu_{1}N_{V}=0,\\ & bN_{V}-\mu_{2}G=0. \end{array}\right)$$

Then, we have $N_{V}=\frac{\alpha }{\mu_{1}}J,G=\frac{\alpha b}{\mu_{1}\mu_{2}}J$. From this,

$$\frac{\sigma N_{V}^{2}}{N_{V}+G+\gamma }=\frac{\frac{\alpha^{2}}{\mu_{1}^{2}}\sigma J^{2}}{\frac{\alpha }{\mu_{1}}J+\frac{\alpha b}{\mu_{1}\mu_{2}}J+\gamma }=\frac{\sigma \mu_{2}\alpha^{2}J^{2}}{\mu_{1}\mu_{2}\alpha J+\mu_{1}\alpha bJ+\mu_{1}^{2}\mu_{2}\gamma }=\alpha J+\left(d_{0}+d_{1}J\right)J.$$

Above equation reduces to:

$$\frac{\sigma \mu_{2}\alpha^{2}J}{\left(\mu_{2}\alpha +\alpha b\right)J+\mu_{1}\mu_{2}\gamma }-\mu_{1}\left(\alpha +d_{0}+d_{1}J\right)=0,$$

further we get

(4) $$N\left(J\right)=A_{1}J^{2}+A_{2}J+A_{3}=0,$$

and

$$\begin{array}{ll} & A_{1}=\mu_{1}d_{1}\left(\mu_{2}\alpha +\alpha b\right)>0,\\ & A_{2}=\mu_{1}\left(d_{0}+\alpha \right)\left(\mu_{2}\alpha +\alpha b\right)+\mu_{1}^{2}\mu_{2}d_{1}\gamma -\sigma \mu_{2}\alpha^{2}=\mu_{1}\alpha \left(d_{0}+\alpha \right)\left(b-b_{p}\right),\\ & A_{3}=\mu_{1}^{2}\mu_{2}\gamma \left(d_{0}+\alpha \right)>0, \end{array}$$

where,

(5) $$b_{p}=\frac{\sigma \mu_{2}\alpha^{2}-\mu_{1}^{2}\mu_{2}d_{1}\gamma }{\mu_{1}\alpha \left(d_{0}+\alpha \right)}-\mu_{2}=\frac{\mu_{1}\mu_{2}\left(\mu_{1}d_{1}\gamma +\alpha \left(d_{0}+\alpha \right)\right)\left(r_{p}-1\right)}{\mu_{1}\alpha \left(d_{0}+\alpha \right)},$$

(6) $$r_{p}=\frac{\sigma \alpha^{2}}{\mu_{1}\left(\mu_{1}d_{1}\gamma +\alpha \left(d_{0}+\alpha \right)\right)}.$$

We can find that when *r*_*p*_ ≤ 1 or *r*_*p*_ > 1 and *b* ≥ *b*_*p*_, all the roots of Eq. (4) are negative, i.e. model (3) has no positive equilibrium points. However, when *r*_*p*_ > 1 and *b* < *b*_*p*_, let $\Delta_{p}=A_{2}^{2}-4A_{1}A_{3}$,

(i) If Δ_*p*_ = 0, for model (3) there exists a positive equilibrium point ${\bar{E}}_{0}^{*}=\left(J_{0}^{*},N_{V0}^{*},G_{0}^{*}\right)$, where,

$$J_{0}^{*}=-\frac{A_{2}}{2A_{1}}=-\frac{\mu_{1}\left(d_{0}+\alpha \right)\left(\mu_{2}\alpha +\alpha b\right)+\mu_{1}^{2}\mu_{2}d_{1}\gamma -\sigma \mu_{2}\alpha^{2}}{2\mu_{1}d_{1}\left(\mu_{2}\alpha +\alpha b\right)},N_{V0}^{*}=\frac{\alpha }{\mu_{1}}J_{0}^{*},G_{0}^{*}=\frac{\alpha b}{\mu_{1}\mu_{2}}J_{0}^{*}.$$

(ii) If Δ_*p*_ < 0, there is no positive equilibrium for model (3).
(iii) If Δ_*p*_ > 0, there exist two positive equilibrium points ${\bar{E}}_{1}^{*}=\left(J_{1}^{*},N_{V1}^{*},G_{1}^{*}\right)$ and ${\bar{E}}_{2}^{*}=\left(J_{2}^{*},N_{V2}^{*},G_{2}^{*}\right)$ for model (3), where,

$$J_{1,2}^{*}=\frac{-A_{2}\mp \sqrt{A_{2}^{2}-4A_{1}A_{3}}}{2A_{1}},N_{V_{1,2}}^{*}=\frac{\alpha }{\mu_{1}}J_{1,2}^{*},G_{1,2}^{*}=\frac{\alpha b}{\mu_{1}\mu_{2}}J_{1,2}^{*}.$$

Therefore, we can obtain the following results:

Lemma 2

(1) *When* *r*_*p*_ ≤ 1 *or if* *r*_*p*_ > 1 *and* *b* ≥ *b*_*p*_ *or if* *r*_*p*_ > 1*,b* < *b*_*p*_ *and* Δ_*p*_ < 0*, model* (3) *only has one zero-equilibrium point* ${\bar{E}}_{0}=\left(0,0,0\right)$*, and no positive equilibrium point.*
(2) *When* *r*_*p*_ > 1*,b* < *b*_*p*_ *and* Δ_*p*_ = 0*, model* (3) *has one zero equilibrium point* ${\bar{E}}_{0}=\left(0,0,0\right)$ *and a positive equilibrium point* ${\bar{E}}_{0}^{*}=\left(J_{0}^{*},N_{V0}^{*},G_{0}^{*}\right)$*.*
(3) *When* *r*_*p*_ > 1*,b* < *b*_*p*_ *and* Δ_*p*_ > 0*, for model* (3)*, there exists one zero equilibrium point* ${\bar{E}}_{0}=\left(0,0,0\right)$ *and two positive equilibrium points* ${\bar{E}}_{1}^{*}=\left(J_{1}^{*},N_{V1}^{*},G_{1}^{*}\right)$*,* ${\bar{E}}_{2}^{*}=\left(J_{2}^{*},N_{V2}^{*},G_{2}^{*}\right)$*, here,* $J_{1}^{*}<J_{2}^{*},N_{V1}^{*}<N_{V2}^{*}$*.*

Stability of these equilibria will be given by the following theorem:

Theorem 1 *Suppose that model* (3) *satisfies the initial conditions are non-zero, and* *J*(0) ≥ 0, *N*_*V*_(0) ≥ 0 *and* *G*(0) ≥ 0*, then the following conclusions hold (Proof: See* Appendix A*):*

(1) *The zero equilibrium point* ${\bar{E}}_{0}$ *of model* (3) *is always locally asymptotically stable.*
(2) *When* *r*_*p*_ > 1*,b* < *b*_*p*_ *and* Δ_*p*_ = 0*, the positive equilibrium point* ${\bar{E}}_{0}^{*}$ *of model* (3) *is locally asymptotically stable.*
(3) *When* *r*_*p*_ > 1*,b* < *b*_*p*_ *and* Δ_*p*_ > 0*, the positive equilibrium point* ${\bar{E}}_{1}^{*}$ *of the model* (3) *is an unstable saddle point.*
(4) *When* *r*_*p*_ > 1*,b* < *b*_*p*_ *and* Δ_*p*_ > 0*, while satisfying* *μ*_2_ ≥ *μ*_1_*, the positive equilibrium point of model* (3) ${\bar{E}}_{2}^{*}$ *is locally asymptotically stable.*

In particular, if no sterile mosquitoes are released (i.e., *G* = 0), and the Allee effect is disregarded (the Allee effect constant *γ* = 0), we have

(7) $$\left\{\begin{array}{ll} & \frac{dJ}{dt}=\sigma N_{V}-\alpha J-\left(d_{0}+d_{1}J\right)J,\\ & \frac{dN_{V}}{dt}=\alpha J-\mu_{1}N_{V}. \end{array}\right)$$

It is easy to find that model (7) has a zero equilibrium point ${\tilde{E}}_{0}=\left(0,0\right)$ and a positive equilibrium point ${\tilde{E}}_{0}^{*}=\left(J^{*},N_{V}^{*}\right)$, where $N_{V}^{*}=\frac{\alpha }{\mu_{1}}J,J^{*}=\frac{\left(\alpha +d_{0}\right)\left(\tilde{r_{p}}-1\right)}{d_{1}}$. Here, we define $\tilde{r_{p}}=\frac{\sigma \alpha }{\mu_{1}\left(\alpha +d_{0}\right)}$. Obviously, $\tilde{r_{p}}$ is the special case of Eq. (6) regarding the *r*_*p*_ expression with *γ* = 0.

Theorem 2 *For model* (7)*, if* $\tilde{r_{p}}\geq 1$*, the zero equilibrium point* ${\tilde{E}}_{0}$ *is unstable, and the positive equilibrium point* ${\tilde{E}}_{0}^{*}$ *is globally asymptotically stable (Proof are given in* Appendix B*).*

**Fig. 1:**
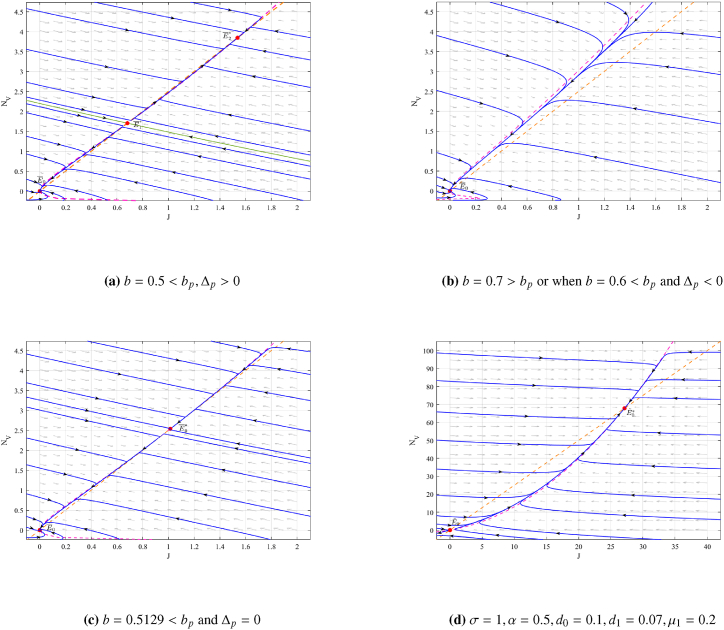
The phase portraits of model (3) and (7).

Here, we illustrate the dynamical behavior of model (3) and model (7) through numerical examples. Using the parameters *σ* = 1, *γ* = 1, *α* = 0.5, *d*_0_ = 0.1, *d*_1_ = 0.07, *μ*_1_ = 0.2, and *μ*_2_ = 0.22, we calculate *b*_*p*_ = 0.6864. The existence of equilibrium points and their asymptotic behavior are depicted in Fig. 1. In Fig. 1(a), when *b* = 0.5 < *b*_*p*_ and Δ_*p*_ > 0, the equilibrium point ${\bar{E}}_{1}^{*}=\left(0.68,1.71\right)$ is an unstable saddle point, while ${\bar{E}}_{2}^{*}=\left(1.54,3.85\right)$ is a stable node. Additionally, the zero equilibrium point ${\bar{E}}_{0}=\left(0,0\right)$ is also a stable node. Fig. 1(b) demonstrates that ${\bar{E}}_{0}=\left(0,0\right)$ remains a stable node when *b* = 0.7 > *b*_*p*_ or when *b* = 0.6 < *b*_*p*_ and Δ_*p*_ < 0. In Fig. 1(c), for *b* = 0.5129 < *b*_*p*_ and Δ_*p*_ = 0, the equilibrium point ${\bar{E}}_{0}^{*}=\left(1.01,2.54\right)$ is a stable node, while the zero equilibrium point ${\bar{E}}_{0}=\left(0,0\right)$ also remains a stable node. Fig. 1(d) illustrates the dynamical behavior of model (7) with the parameters *σ* = 1, *α* = 0.5, *d*_0_ = 0.1, *d*_1_ = 0.07, and *μ*_1_ = 0.2. Here, ${\tilde{E}}_{0}=\left(0,0\right)$ is an unstable saddle point, while ${\tilde{E}}_{0}^{*}=\left(27.14,67.86\right)$ is a stable node.

## 4 Analysis of model considering virus distribution

In this section, we consider the case of *G* ≠ 0. According to the discussion in Section 3, when the positive equilibrium of model (3) is stabilised, the following condition holds:

(8) $$\underset{t\to \infty }{\lim}J\left(t\right)=J^{*},J^{*}=\left\{\begin{array}{l} J_{2}^{*},r_{p}>1,b<b_{p},\Delta_{p}>0,\;\\ J_{0}^{*},r_{p}>1,b<b_{p},\Delta_{p}=0.\; \end{array}\right)$$

(9) $$\underset{t\to \infty }{\lim}N_{V}\left(t\right)=N_{V}^{*},N_{V}^{*}=\left\{\begin{array}{l} N_{V2}^{*},J^{*}=J_{2}^{*},\mu_{2}\geq \mu_{1},\;\\ N_{V0}^{*},J^{*}=J_{0}^{*}.\; \end{array}\right)$$

(10) $$\underset{t\to \infty }{\lim}G\left(t\right)=G^{*},G^{*}=\left\{\begin{array}{l} G_{2}^{*},J^{*}=J_{2}^{*},\mu_{2}\geq \mu_{1},\;\\ G_{0}^{*},J^{*}=J_{0}^{*}.\; \end{array}\right)$$

In Theorem 1, since ${\bar{E}}_{1}^{*}$ is unstable, for model (2), we will no longer consider the equilibrium point corresponding to ${\bar{E}}_{1}^{*}$.

### 4.1 The basic reproduction number and disease-free equilibrium point

The dynamical behaviour of model (2) is equivalent to the following model:

(11) $$\left\{\begin{array}{ll} & \frac{dS_{H}}{dt}=\Lambda -r\beta_{H}I_{V}S_{H}-\mu_{H}S_{H},\\ & \frac{dI_{H}}{dt}=r\beta_{H}I_{V}S_{H}-\left(\mu_{H}+\delta +\eta \right)I_{H},\\ & \frac{dI_{V}}{dt}=\alpha I_{J}+r\beta_{V}I_{H}\left(N_{V}-I_{V}\right)-\mu_{1}I_{V},\\ & \frac{dI_{J}}{dt}=\beta \left(J-I_{J}\right)V-\alpha I_{J}-\left(d_{0}+d_{1}J\right)I_{J},\\ & \frac{dV}{dt}=\theta I_{H}-\mu V,\\ & \frac{dJ}{dt}=\frac{\sigma N_{V}}{N_{V}+G+\gamma }N_{V}-\alpha J-\left(d_{0}+d_{1}J\right)J,\\ & \frac{dN_{V}}{dt}=\alpha J-\mu_{1}N_{V},\\ & \frac{dG}{dt}=bN_{V}-\mu_{2}G. \end{array}\right)$$

This clearly indicates the existence of a disease-free equilibrium point $E_{0}=\left(\frac{\Lambda }{\mu_{H}},0,0,0,0,J^{*},N_{V}^{*},G^{*}\right)$. Next, using the regenerative matrix method (Van et al., 2002), we can obtain:

$$F=\left(\begin{array}{llll} 0 & \frac{r\beta_{H}\Lambda }{\mu_{H}} & 0 & 0\\ r\beta_{V}N_{V}^{*} & 0 & 0 & 0\\ 0 & 0 & 0 & \beta J^{*}\\ 0 & 0 & 0 & 0 \end{array}\right),V=\left(\begin{array}{llll} \mu_{H}+\delta +\eta & 0 & 0 & 0\\ 0 & \mu_{1} & -\alpha & 0\\ 0 & 0 & \alpha +d_{0}+d_{1}J^{*} & 0\\ -\theta & 0 & 0 & \mu \end{array}\right).$$

Furthermore

(12) $$FV^{-1}=\left(\begin{array}{llll} 0 & \frac{r\beta_{H}\Lambda }{\mu_{1}\mu_{H}} & \frac{\alpha r\beta_{H}\Lambda }{\mu_{1}\mu_{H}\left(\alpha +d_{0}+d_{1}J^{*}\right)} & 0\\ \frac{r\beta_{V}N_{V}^{*}}{\left(\mu_{H}+\delta +\eta \right)} & 0 & 0 & 0\\ \frac{\theta \beta J^{*}}{\mu \left(\mu_{H}+\delta +\eta \right)} & 0 & 0 & \frac{\beta J^{*}}{\mu }\\ 0 & 0 & 0 & 0 \end{array}\right),$$

then the basic reproduction number of model (11) is

(13) $$R_{0}=\sqrt{R_{01}+R_{02}},$$

where,

$$R_{01}=\frac{r^{2}\beta_{H}\beta_{V}\Lambda N_{V}^{*}}{\mu_{H}\mu_{1}\left(\mu_{H}+\delta +\eta \right)},R_{02}=\frac{\alpha r\beta_{H}\Lambda \theta \beta J^{*}}{\mu \mu_{H}\mu_{1}\left(\mu_{H}+\delta +\eta \right)\left(\alpha +d_{0}+d_{1}J^{*}\right)}.$$

Theorem 3 *If* *R*_0_ ≤ 1 *and the condition (4) in* Theorem 1 *is satisfied, the disease-free equilibrium* *E*_0_ *of model* (11) *is locally asymptotically stable. Conversely, when* *R*_0_ > 1*, the disease-free equilibrium* *E*_0_ *is unstable.*

***Proof*.** *The Jacobian matrix of model (11) at the disease-free equilibrium point* *E*_0_ *is*

$$J\left(E_{0}\right)=\left(\begin{array}{ll} J_{2} & 0\\ 0 & J_{1}\left({\bar{E}}^{*}\right) \end{array}\right),J_{1}\left({\bar{E}}^{*}\right)=\left\{\begin{array}{l} J_{1}\left({\bar{E}}_{0}^{*}\right),J^{*}=J_{0}^{*},\;\\ J_{1}\left({\bar{E}}_{2}^{*}\right),J^{*}=J_{2}^{*},\; \end{array}\right)$$

*where*

$$J_{2}=\left(\begin{array}{lllll} -\mu_{H} & 0 & \frac{-r\beta_{H}\Lambda }{\mu_{H}} & 0 & 0\\ 0 & -m & \frac{r\beta_{H}\Lambda }{\mu_{H}} & 0 & 0\\ 0 & r\beta_{V}N_{V}^{*} & -\mu_{1} & \alpha & 0\\ 0 & 0 & 0 & -\left(\alpha +d_{0}+d_{1}J^{*}\right) & \beta J^{*}\\ 0 & \theta & 0 & 0 & -\mu \end{array}\right),$$

*it follows that the characteristic equation of model (11) at the disease-free equilibrium point* *E*_0_ *is*

$$\left|\begin{array}{l} \lambda E-J_{2} \end{array}\right|=\left|\begin{array}{lllll} \lambda +\mu_{H} & 0 & \frac{r\beta_{H}\Lambda }{\mu_{H}} & 0 & 0\\ 0 & \lambda +m & \frac{-r\beta_{H}\Lambda }{\mu_{H}} & 0 & 0\\ 0 & -r\beta_{V}N_{V}^{*} & \lambda +\mu_{1} & -\alpha & 0\\ 0 & 0 & 0 & \lambda +n & -\beta J^{*}\\ 0 & -\theta & 0 & 0 & \lambda +\mu \end{array}\right|=0.$$

*Clearly, one of the eigenvalues is* *λ*_1_ = *μ*_*H*_*, and the remaining eigenvalues are the roots of the following equation*

(14) $$p\left(\lambda \right)=\lambda^{4}+a_{1}\lambda^{3}+a_{2}\lambda^{2}+a_{3}\lambda +a_{4}=0,$$

*where*,

$$\begin{array}{ll} & a_{1}=m+\mu_{1}+n+\mu ,\\ & a_{2}=\left(n+\mu \right)\left(m+\mu_{1}\right)+n\mu +n\mu \left(m+\mu_{1}\right)+\mu_{1}m-\frac{r^{2}\beta_{H}\beta_{V}\Lambda N_{V}^{*}}{\mu_{H}}\\ & =\left(n+\mu \right)\left(m+\mu_{1}\right)+n\mu +n\mu \left(m+\mu_{1}\right)+\mu_{1}m\left(1-R_{01}\right),\\ & a_{3}=\left(n+\mu \right)\left(m\mu_{1}-\frac{r^{2}\beta_{H}\beta_{V}\Lambda N_{V}^{*}}{\mu_{H}}\right)=m\mu_{1}\left(n+\mu \right)\left(1-R_{01}\right),\\ & a_{4}=\frac{n\mu m\mu_{1}\mu_{H}-\left(r^{2}\beta_{H}\beta_{V}\Lambda N_{V}^{*}n\mu +\alpha r\beta_{H}\Lambda \theta \beta J^{*}\right)}{\mu_{H}}=n\mu m\mu_{1}\left(1-R_{0}^{2}\right). \end{array}$$

Then, *we have*

$$\begin{array}{ll} & H_{1}=a_{1}=m+\mu_{1}+n+\mu >0,\\ & H_{2}=\left|\begin{array}{ll} a_{1} & a_{3}\\ 1 & a_{2} \end{array}\right|=a_{1}a_{2}-a_{3}=\left(m+\mu_{1}\right)\left[\left(n+\mu \right)\left(m+\mu_{1}\right)+n\mu +n\mu \left(m+\mu_{1}\right)+m\mu_{1}\left(1-R_{01}\right)\right]\\ & +\left(n+\mu \right)\left[\left(n+\mu \right)\left(m+\mu_{1}\right)+n\mu +n\mu \left(m+\mu_{1}\right)\right],\\ & H_{3}=\left|\begin{array}{lll} a_{1} & a_{3} & 0\\ 1 & a_{2} & a_{4}\\ 0 & a_{1} & a_{3} \end{array}\right|=a_{3}\left(a_{1}a_{2}-a_{3}\right)-a_{1}^{2}a_{4}\\ & =\mu_{1}^{2}m^{2}{\left(1-R_{01}\right)}^{2}\left(n+\mu \right)\left(m+\mu_{1}\right)+n\mu m\mu_{1}\left(n+\mu \right)\left(m+\mu_{1}\right)\left[\left(m+\mu_{1}\right)+{\left(n+\mu \right)}^{2}\right]\left(1-R_{01}\right)\\ & +m\mu_{1}\left(n^{2}+\mu^{2}+n\mu \right)\left(m+\mu_{1}\right)\left(m+\mu_{1}+n+\mu \right)\left(1-R_{01}\right)+{\left(m+\mu_{1}+n+\mu \right)}^{2}n\mu m\mu_{1}R_{02}. \end{array}$$

*When* *R*_0_ < 1*,* $R_{0}^{2}<1$*,* *R*_01_ < 1*, and* *R*_02_ < 1*, it follows that* *a*_*i*_ > 0 *(for* *i* = 1, 2, 3, 4*) and* *H*_*i*_ > 0 *(for* *i* = 1, 2, 3, 4*). Therefore, according to the Hurwitz criterion* (Ma et al., 2015)*, all the eigenvalues of* *J*_2_ *have negative real parts. That means model (11) is locally asymptotically stable at the disease-free equilibrium point* *E*_0_.

Next, we consider the global stability of model (11). Substituting Eq. (8), Eq. (9) and Eq. (10) into model (11), we get

(15) $$\left\{\begin{array}{ll} & \frac{dS_{H}}{dt}=\Lambda -r\beta_{H}I_{V}S_{H}-\mu_{H}S_{H},\\ & \frac{dI_{H}}{dt}=r\beta_{H}I_{V}S_{H}-\left(\mu_{H}+\delta +\eta \right)I_{H},\\ & \frac{dI_{V}}{dt}=\alpha I_{J}+r\beta_{V}I_{H}\left(N_{V}^{*}-I_{V}\right)-\mu_{1}I_{V},\\ & \frac{dI_{J}}{dt}=\beta \left(J^{*}-I_{J}\right)V-\alpha I_{J}-\left(d_{0}+d_{1}J^{*}\right)I_{J},\\ & \frac{dV}{dt}=\theta I_{H}-\mu V. \end{array}\right)$$

The model (15) is the limiting model of model (11)(Castillo-Chavez & Thieme, 1994). We present the following results:

Theorem 4 *If* *R*_0_ < 1 *and condition (4) in* Theorem 1 *is also satisfied, then model* (15) *is globally asymptotically stable at the disease-free equilibrium point* $E_{0}^{*}=\left(\frac{\Lambda }{\mu_{H}},0,0,0,0\right)$*.*

***Proof*.** Theorem 3 *proves the local stability of* *E*_0_*, now we only prove the global attractivity of* $E_{0}^{*}$. *For the positive solutions* (*S*_*H*_, *I*_*H*_, *I*_*V*_, *I*_*J*_, *V*) *of model (15), we have*

(16) $$\left\{\begin{array}{ll} & \frac{dI_{H}}{dt}\leq r\beta_{H}I_{V}\frac{\Lambda }{\mu_{H}}-\left(\mu_{H}+\delta +\eta \right)I_{H},\\ & \frac{dI_{V}}{dt}\leq \alpha I_{J}+r\beta_{V}I_{H}\left(N_{V}^{*}-I_{V}\right)-\mu_{1}I_{V},\\ & \frac{dI_{J}}{dt}\leq \beta \left(J^{*}-I_{J}\right)V-\alpha I_{J}-\left(d_{0}+d_{1}J^{*}\right)I_{J},\\ & \frac{dV}{dt}\leq \theta I_{H}-\mu V. \end{array}\right)$$

We also modelled the following

(17) $$\{\begin{array}{ll} & \frac{d\tilde{I_{H}}}{dt}=r\beta_{H}\tilde{I_{V}}\frac{\Lambda }{\mu_{H}}-\left(\mu_{H}+\delta +\eta \right)\tilde{I_{H}},\\ & \frac{d\tilde{I_{V}}}{dt}=\alpha \tilde{I_{J}}+r\beta_{V}\tilde{I_{H}}\left(N_{V}^{*}-\tilde{I_{V}}\right)-\mu_{1}\tilde{I_{V}},\\ & \frac{d\tilde{I_{J}}}{dt}=\beta \left(J^{*}-\tilde{I_{J}}\right)\tilde{V}-\alpha \tilde{I_{J}}-\left(d_{0}+d_{1}J^{*}\right)\tilde{I_{J}},\\ & \frac{d\tilde{V}}{dt}=\theta \tilde{I_{H}}-\mu \tilde{V}. \end{array}$$

*Obviously, model (16) is a non-degenerate linear cooperative model with stability at the origin* (0, 0, 0, 0) *completely determined by the Jacobi matrix* *J*_0_ = *F* − *V, where* *F* *and* *V* *are given by the* equation (12). *According to the result in* Theorem 2 Van et al. (2002) *and the results of the basic reproduction number, model (17) is stable at* (0, 0, 0, 0) *if* *R*_0_ < 1*. That is, for* *R*_0_ < 1*, when* *t* → +*∞,*

$$\tilde{I_{H}}\to 0,\tilde{I_{V}}\to 0,\tilde{I_{J}}\to 0,\tilde{V}\to 0.$$

*Using the principle of comparison, we can get: if* *t* → +*∞,*

(18) $$I_{H}\to 0,I_{V}\to 0,I_{J}\to 0,V\to 0.$$

*Substituting* Eq. (18) i*nto model (15), we get* $\underset{t\to +\infty }{\lim}\frac{dS_{H}}{dt}+\mu_{H}S_{H}=\Lambda$*, hence: when* *t* → +*∞,* $S_{H}\to \frac{\Lambda }{\mu_{H}}$*.*

*According to the theory of limit system* (Castillo-Chavez & Thieme, 1994)*, for model (15), the disease-free equilibrium* $E_{0}^{*}=\left(\frac{\Lambda }{\mu_{H}},0,0,0,0,0\right)$ *is a global attractor if* *R*_0_ < 1*, thus proving* Theorem 4.

### 4.2 The endemic disease equilibrium

In this subsection, we will establish some conditions for the existence of the endemic equilibrium point. To this end, the endemic equilibrium point $E^{*}=\left(S_{H}^{*},I_{H}^{*},I_{V}^{*},I_{J}^{*},V^{*},J^{*},N_{V}^{*},G^{*}\right)$ of model (11), where the right-hand side of model (15) is zero, can be determined by the following equation:

(19) $$\left\{\begin{array}{ll} & \Lambda -r\beta_{H}I_{V}^{*}S_{H}^{*}-\mu_{H}S_{H}^{*}=0,\\ & r\beta_{H}I_{V}^{*}S_{H}^{*}-\left(\mu_{H}+\delta +\eta \right)I_{H}^{*}=0,\\ & \alpha I_{J}^{*}+r\beta_{V}I_{H}^{*}\left(N_{V}^{*}-I_{V}^{*}\right)-\mu_{1}I_{V}^{*}=0,\\ & \beta \left(J^{*}-I_{J}^{*}\right)V^{*}-\alpha I_{J}^{*}-\left(d_{0}+d_{1}J^{*}\right)I_{J}^{*}=0,\\ & \theta I_{H}^{*}-\mu V^{*}=0. \end{array}\right)$$

Let *m* = *μ*_*H*_ + *δ* + *η*, *n* = *α* + *d*_0_ + *d*_1_*J*∗, *p*_1_ = *rβ*_*H*_, *p*_2_ = *rβ*_*V*_, from Eq. (19),

$$S_{H}^{*}=\frac{\Lambda }{p_{1}I_{V}^{*}+\mu_{H}},V^{*}=\frac{\Lambda \theta p_{1}I_{V}^{*}}{\mu m\left(p_{1}I_{V}^{*}+\mu_{H}\right)},I_{H}^{*}=\frac{p_{1}I_{V}^{*}S_{H}^{*}}{m}=\frac{\Lambda p_{1}I_{V}^{*}}{m\left(p_{1}I_{V}^{*}+\mu_{H}\right)},$$

(20) $$I_{J}^{*}=\frac{\beta J^{*}\Lambda \theta p_{1}I_{V}^{*}}{n\mu m\left(p_{1}I_{V}^{*}+\mu_{H}\right)+\beta \Lambda \theta p_{1}I_{V}^{*}}.$$

In the third equation of Eq. (19) gives

(21) $$I_{J}^{*}=\frac{\mu_{1}I_{V}^{*}-p_{2}I_{H}^{*}\left(N_{V}^{*}-I_{V}^{*}\right)}{\alpha }=\frac{\mu_{1}m\left(p_{1}I_{V}^{*}+\mu_{H}\right)I_{V}^{*}-p_{2}\left(N_{V}^{*}-I_{V}^{*}\right)\Lambda p_{1}I_{V}^{*}}{\alpha m\left(p_{1}I_{V}^{*}+\mu_{H}\right)}.$$

By combining Eq. (20) and Eq. (21), $I_{V}^{*}$ satisfies the following equation

(22) $$C_{1}{\left(I_{V}^{*}\right)}^{2}+C_{2}I_{V}^{*}+C_{3}=0,$$

here,

$$\begin{array}{ll} & C_{1}=\left(n\mu mp_{1}+\beta \Lambda \theta p_{1}\right)\left(p_{1}\mu_{1}m+p_{1}p_{2}\Lambda \right)>0,\\ & C_{2}=\left(n\mu mp_{1}+\beta \Lambda \theta p_{1}\right)\left(\mu_{1}m\mu_{H}-p_{1}p_{2}\Lambda N_{V}^{*}\right)+n\mu m\mu_{H}\left(\mu_{1}mp_{1}+p_{1}p_{2}\Lambda \right)-m\alpha p_{1}^{2}\Lambda \theta \beta J^{*},\\ & =\mu_{1}m\mu_{H}p_{1}\left[n\mu m\left(\frac{p_{2}\Lambda +2\mu_{1}m}{\mu_{1}m}-R_{0}^{2}\right)+\beta \Lambda \theta \left(1-R_{01}\right)\right]\\ & C_{3}=m\mu_{H}n\mu \left(\mu_{1}m\mu_{H}-p_{1}p_{2}N_{V}^{*}\Lambda \right)-m\mu_{H}\alpha p_{1}\Lambda \theta \beta J^{*}\\ & =m\mu_{H}\left[n\mu \mu_{1}m\mu_{H}-\left(n\mu p_{1}p_{2}\Lambda N_{V}^{*}+\alpha p_{1}\Lambda \theta \beta J^{*}\right)\right]=n\mu \mu_{1}m^{2}\mu_{H}^{2}\left(1-R_{0}^{2}\right). \end{array}$$

Therefore, we can express each of the two roots of Eq. (22) as

(23) $$I_{V1}^{*}=\frac{-C_{2}+\sqrt{C_{2}^{2}-4C_{1}C_{3}}}{2C_{1}},I_{V2}^{*}=\frac{-C_{2}-\sqrt{C_{2}^{2}-4C_{1}C_{3}}}{2C_{1}}.$$

For model (11), there exists an endemic equilibrium point if and only if there exists a positive root of Eq. (22). We obtain the existence of roots of Eq. (22) by analysing the following 3 cases:

(i) When *R*_0_ > 1, it means that *C*_3_ < 0, then $I_{V1}^{*}I_{V2}^{*}=\frac{C_{3}}{C_{1}}<0$, and hence there exists only positive root of Eq. (22), $I_{V}^{*}=I_{V1}^{*}$.
(ii) When *R*_0_ = 1, this implies that *C*_3_ = 0, *C*_2_ > 0, and Eq. (22) is written as $C_{1}{\left(I_{V}^{*}\right)}^{2}+C_{2}I_{V}^{*}=0$, then $I_{V}^{*}=-\frac{C_{2}}{C_{1}}<0$, and hence Eq. (22) has no positive roots.
(iii) When *R*_0_ < 1, which entails *C*_3_ > 0, *C*_2_ > 0, then $I_{V1}^{*}+I_{V2}^{*}=-\frac{C_{2}}{C_{1}}<0$, $I_{V1}^{*}I_{V2}^{*}=\frac{C_{3}}{C_{1}}>0$, and therefore there are no positive roots of Eq (22).

Based on the aforementioned analyses, for model (11), the following conclusions are presented regarding the existence of endemic equilibrium points:

Theorem 5 *For model* (11)*, if condition (4) in* Theorem 1 *is satisfied, the following holds:*

(1) *If* *R*_0_ > 1*, then there exists a unique endemic equilibrium* $E^{*}=\left(S_{H}^{*},I_{H}^{*},I_{V}^{*},I_{J}^{*},V,J^{*},N_{V}^{*},G^{*}\right)$ *of model* (11)*, where* $I_{V}^{*}=I_{V1}^{*}$*;*
(2) *If* *R*_0_ ≤ 1 *and* *C*_2_ > 0*, then there is no endemic equilibrium for model* (11).

Next, we will consider the stability of model (11)at the endemic equilibrium point *E*∗. According Eq. (20) and Eq. (21), let *f*(*I*_*V*_) = *f*_1_(*I*_*V*_) − *f*_2_(*I*_*V*_), here

(24) $$f_{1}\left(I_{V}\right)=\frac{\left(p_{1}I_{V}+\mu_{H}\right)\mu_{1}mI_{V}-p_{1}p_{2}\Lambda I_{V}\left(N_{V}^{*}-I_{V}\right)}{\alpha m\left(p_{1}I_{V}+\mu_{H}\right)},$$

(25) $$f_{2}\left(I_{V}\right)=\frac{\beta J^{*}\Lambda \theta p_{1}I_{V}}{n\mu m\left(p_{1}I_{V}+\mu_{H}\right)+\beta \Lambda \theta p_{1}I_{V}}.$$

then

(26) $$f^{\prime }\left(I_{V}\right)=f_{1}^{\prime }\left(I_{V}\right)-f_{2}^{\prime }\left(I_{V}\right),$$

where,

(27) $$f_{1}^{\prime }\left(I_{V}\right)=\frac{\left[p_{1}p_{2}\Lambda I_{V}+\mu_{1}m\left(p_{1}I_{V}+\mu_{H}\right)\right]\alpha m\left(p_{1}I_{V}+\mu_{H}\right)-\alpha m\mu_{H}p_{1}p_{2}\Lambda \left(N_{V}^{*}-I_{V}\right)}{{\left[\alpha m\left(p_{1}I_{V}+\mu_{H}\right)\right]}^{2}},$$

(28) $$f_{2}^{\prime }\left(I_{V}\right)=\frac{\beta J^{*}\Lambda \theta p_{1}n\mu m\mu_{H}}{{\left[n\mu m\left(p_{1}I_{V}+\mu_{H}\right)+\beta \Lambda \theta p_{1}I_{V}\right]}^{2}}.$$

According to Eq. (22) and Eq. (23), we can see that *f*(*I*_*V*_) can also be expressed as

(29) $$f\left(I_{V}\right)=f_{1}\left(I_{V}\right)-f_{2}\left(I_{V}\right)=I_{V}\left(I_{V}-I_{V1}^{*}\right)\left(I_{V}-I_{V2}^{*}\right),$$

therefore $f^{\prime }\left(I_{V1}^{*}\right)=I_{V1}^{*}\left(I_{V1}^{*}-I_{V2}^{*}\right)>0$.

Theorem 6 *If* *R*_0_ > 1*, condition (4) in* Theorem 1 *is also satisfied, then the only endemic equilibrium point* *E*∗ *of model* (11) *is locally asymptotically stable, or unstable dimension and the number of central manifolds are both even.*

***Proof*.** *The Jacobian matrix of model (11) at the endemic equilibrium point* *E*∗ *is*

(30) $$J\left(E^{*}\right)=\left(\begin{array}{ll} J_{3} & 0\\ 0 & J_{1}\left({\bar{E}}^{*}\right) \end{array}\right),$$

(31) $$J_{3}=\left(\begin{array}{lllll} -\mu_{H}-r\beta_{H}I_{V}^{*} & 0 & -r\beta_{H}S_{H}^{*} & 0 & 0\\ r\beta_{H}I_{V}^{*} & -m & r\beta_{H}S_{H}^{*} & 0 & 0\\ 0 & r\beta_{V}\left(N_{V}^{*}-I_{V}^{*}\right) & -r\beta_{V}I_{H}^{*}-\mu_{1} & \alpha & 0\\ 0 & 0 & 0 & -\beta V^{*}-n & \beta \left(J^{*}-I_{J}^{*}\right)\\ 0 & \theta & 0 & 0 & -\mu \end{array}\right),$$

*The characteristic equation is*

$$\left|\begin{array}{l} \lambda E-J_{3} \end{array}\right|=\left|\begin{array}{lllll} \lambda +\left(\mu_{H}+k_{1}\right) & 0 & k_{2} & 0 & 0\\ -k_{1} & \lambda +m & -k_{2} & 0 & 0\\ 0 & -k_{4} & \lambda +\left(\mu_{1}+k_{3}\right) & -\alpha & 0\\ 0 & 0 & 0 & \lambda +k_{5} & -k_{6}\\ 0 & -\theta & 0 & 0 & \lambda +\mu \end{array}\right|=0,$$

*where*

$$k_{1}=p_{1}I_{V}^{*},k_{2}=p_{1}S_{H}^{*},k_{3}=p_{2}I_{H}^{*},k_{4}=p_{2}\left(N_{V}^{*}-I_{V}^{*}\right),k_{5}=\beta V^{*}+n,k_{6}=\beta \left(J^{*}-I_{J}^{*}\right).$$

Moreover, *the characteristic equation is given by*

(32) $$\lambda^{5}+c_{1}\lambda^{4}+c_{2}\lambda^{3}+c_{3}\lambda^{2}+c_{4}\lambda +c_{5}=0.$$

*here*,

$$\begin{array}{ll} & c_{1}=k_{1}+k_{3}+k_{5}+\mu_{1}+\mu_{H}+m+\mu >0,\\ & c_{2}=mk_{5}+\left(k_{1}+k_{3}+\mu_{H}+\mu_{1}\right)\left(k_{5}+\mu +m\right)+\left(k_{1}+\mu_{H}\right)\left(k_{3}+\mu_{1}\right)-k_{2}k_{4},\\ & c_{3}=\mu \left(k_{5}+m\right)+k_{5}\mu m+k_{5}m\left(k_{1}+k_{3}+\mu_{H}+\mu_{1}\right)\\ & +\left(k_{1}+\mu_{H}\right)\left(k_{3}+\mu_{1}\right)\left(k_{5}+\mu +m\right)-k_{2}k_{4}\mu_{H}-\left(k_{5}+\mu \right)k_{2}k_{4},\\ & c_{4}=\left(k_{1}+k_{3}+\mu_{H}+\mu_{1}\right)\left[\left(k_{5}+m\right)\mu +k_{5}\mu m\right]+k_{5}m\left(k_{1}+\mu_{H}\right)\left(k_{3}+\mu_{1}\right),\\ & c_{5}=\mu mk_{5}\left(k_{1}+\mu_{H}\right)\left(k_{3}+\mu_{1}\right)-\left(\alpha \theta k_{6}+\mu k_{4}k_{5}\right)\mu_{H}k_{2}. \end{array}$$

*From* Eq. (26), Eq. (27) *and* Eq. (28), *we get*

$$c_{5}=\alpha \left[\beta \Lambda \theta p_{1}I_{V}^{*}+n\mu m\left(p_{1}I_{V}^{*}+\mu_{H}\right)\right]f^{\prime }\left(I_{V1}^{*}\right)>0.$$

*Suppose* *λ*_1_*,* *λ*_2_*,* *λ*_3_*,* *λ*_4_*,* *λ*_5_ *are the five roots of* Eq. (32)*, based on the relationship between the roots and coefficients of an algebraic equation,*

$$c_{1}=-\left(\lambda_{1}+\lambda_{2}+\lambda_{3}+\lambda_{4}+\lambda_{5}\right)>0,c_{5}=-\lambda_{1}\lambda_{2}\lambda_{3}\lambda_{4}\lambda_{5}>0.$$

*This would imply that all eigenvalues of the eigenequation* (32) *are negative*, *or that the number of eigenvalues with positive real parts is even*. *The* proof of Theorem 6 *is complete*.

### 4.3 Consistent persistence

Next, we demonstrate that when *R*_0_ > 1, ZIKV persists in the population, and the infectious disease becomes endemic. Therefore, the basic reproduction number *R*_0_ is an essential threshold for disease persistence and extinction.

Theorem 7 *If* *R*_0_ > 1*, as well as satisfying condition (4) in* Theorem 1*, model* (11) *is consistently persistent, i.e. there exists a constant* *ɛ* > 0 *such that for model* (11) *satisfying the initial conditions*

$$x_{0}=\left(S_{H}\left(0\right),I_{H}\left(0\right),I_{V}\left(0\right),I_{J}\left(0\right),V\left(0\right),J\left(0\right),N_{V}\left(0\right),G\left(0\right)\right)$$

*for each solution* *φ*_*t*_(*x*_0_) = (*S*_*H*_(*t*), *I*_*H*_(*t*), *I*_*V*_(*t*), *I*_*J*_(*t*), *V*(*t*), *J*(*t*), *N*_*V*_(*t*), *G*(*t*))*, we have*

$$\underset{t\to +\infty }{\lim}\inf I_{H}\left(t\right)>\epsilon ,\underset{t\to +\infty }{\lim}\inf I_{V}\left(t\right)>\epsilon ,\underset{t\to +\infty }{\lim}\inf I_{J}\left(t\right)>\epsilon ,\underset{t\to +\infty }{\lim}\inf\;V\left(t\right)>\epsilon ,$$

*where* *I*_*H*_(0) + *I*_*V*_(0) + *I*_*J*_(0) + *V*(0) > 0.

***Proof*.** *For* *R*_0_ > 1*, define*

$$\begin{array}{ll} & \Omega_{0}=\left\{\left(S_{H},I_{H},I_{V},I_{J},V,J,N_{V},G\right)\in \Omega |I_{H}>0,I_{V}>0,I_{J}>0,V>0\right\},\\ & \partial \Omega_{0}=\left\{\left(S_{H},I_{H},I_{V},I_{J},V,J,N_{V},G\right)\in \Omega |I_{H}=0,I_{V}=0,I_{J}=0,V=0\right\}. \end{array}$$

*Clearly, both* Ω *and* Ω_0_ *are positively invariant and* *∂*Ω_0_ *is a comparatively closed set of* Ω*. The tightness and positive invariance of* Ω *imply that model (11) is pointwise dissipative. Defining*

$$\Gamma =\left\{x_{0}\in \partial \Omega_{0}:\varphi_{t}\left(x_{0}\right)\in \partial \Omega_{0},\forall t>0\right\},$$

$$D=\left\{\left(S_{H},I_{H},I_{V},I_{J},V,J,N_{V},G\right)\in \Omega |I_{H}=I_{V}=I_{J}=V=0\right\}.$$

*Then we prove that* Γ = *D. Obviously* *D* ⊆Γ ⊆ *∂*Ω_0_*, on the other hand, for any* *x*_0_ ∈ *∂*Ω_0_ ∖ *D, we have* *I*_*H*_(0) + *I*_*V*_(0) + *I*_*J*_(0) + *V*(0) > 0*. From the irreducibility of* *F* − *V, for* *∀t* > 0*, there is* *φ*_*t*_(*x*_0_) ∈ Ω_0_*, and* *x*_0_∉Γ*. Therefore* Γ ⊆ *D, and thus* Γ = *D. From the question, if* *x*_0_ ∈ Γ*, then there is only one limit set* *E*_0_*, so* ${⋃}_{x_{0}\in \Gamma }\omega \left(x_{0}\right)=E_{0}$*. Thus* *E*_0_ *is a compact and isolated invariant set in* Γ.

*Below we prove that* *W*^*S*^(*E*_0_)⋂Ω_0_ = ∅*, where* *W*^*S*^(*E*_0_) *is a stable manifold of* *E*_0_*. Assuming it does not hold, there exists* *x*_0_ ∈ Ω_0_ *such that* $\underset{t\to +\infty }{\lim}\varphi_{t}\left(x_{0}\right)=E_{0}$*. Define*

$$\begin{array}{ll} & M\left(\xi \right)=F-V-\xi G\\ & =\left(\begin{array}{llll} -\left(\mu_{H}+\delta +\eta \right) & r\beta_{H}\left(\frac{\Lambda }{\mu_{H}}-\xi \right) & 0 & 0\\ r\beta_{V}\left(N_{V}^{*}-\xi \right) & -\mu_{1} & \alpha & 0\\ 0 & 0 & -\left(\alpha +d_{0}+d_{1}J^{*}\right) & \beta \left(J^{*}-\xi \right)\\ \theta & 0 & 0 & -\mu \end{array}\right), \end{array}$$

*where*,

$$G=\left(\begin{array}{llll} 0 & r\beta_{H} & 0 & 0\\ r\beta_{V} & 0 & 0 & 0\\ 0 & 0 & 0 & \beta \\ 0 & 0 & 0 & 0 \end{array}\right).$$

*By* Theorem 2 (Van et al., 2002)*, the spectrum of* *F* − *V* *bounds* *s*(*F* − *V*) = *s*(*M*(0)) > 0 *if and only if* *R*_0_ > 1*. Since* *s*(*M*(*ξ*)) *is continuous with respect to* *ξ, there exists a sufficiently small* *ξ*_1_ *such that: when* *ξ* ∈ [0, *ξ*_1_]*,* *s*(*M*(*ξ*)) > 0*. In addition, after a translation transformation, we arrive*

$${\left\|\varphi_{t}\left(x_{0}\right)-E_{0}\right\|}_{2}={\left\|\varphi_{t}\left(x_{0}\right)\right\|}_{2}\leq \xi_{1},\forall t\geq 0,$$

*here*, ${\left\|\cdot \right\|}_{2}$ *is the regular Euclidean paradigm*. *Thus*,

$${\left(\frac{dI_{H}}{dt},\frac{dI_{V}}{dt},\frac{dI_{J}}{dt},\frac{dV}{dt}\right)}^{T}\geq M\left(\xi_{1}\right){\left(I_{H},I_{V},I_{J},V\right)}^{T}.$$

*Because* *M*(*ξ*_1_) *is irreducible and intrinsically non-negative, it follows from the Perron-Frobenius Theorem (*Smith, 1995*) that:* *M*(*ξ*_1_) *has a positive eigenvector associated with* *s*(*M*(*ξ*_1_))*. By comparing the principles (*Smith & Waltman, 1995*),*

$$\underset{t\to +\infty }{\lim}I_{H}\left(t\right)=+\infty ,\underset{t\to +\infty }{\lim}I_{V}\left(t\right)=+\infty ,\underset{t\to +\infty }{\lim}I_{J}\left(t\right)=+\infty ,\underset{t\to +\infty }{\lim}V\left(t\right)=+\infty ,$$

*this contradicts the dissipative nature of model (11). Note that* *E*_0_ *is an isolated invariant set and acyclic, i.e.* *W*^*S*^(*E*_0_)⋂Ω_0_ = ∅.

*Thereby, by Theorem 4.6* Thieme. (1993)*, model (11) is consistently persistent when* *R*_0_ > 1.

## 5 Optimal control strategy

In this section, we discuss the optimal control strategy of ZIKV by considering the control effect and resource constraints comprehensively. The function *u*_1_(*t*) represents the risk of human-mosquito transmission, which can be mitigated through the use of mosquito nets, mosquito coils, repellents, and similar measures. The function *u*_2_(*t*) represents the reduction in mosquito populations through the application of insecticides. The function *u*_3_(*t*) represents the control of mosquito populations by releasing sterile mosquitoes. *u*_4_(*t*) represents the degree of ZIKV transmission control achieved through public education and awareness campaigns, which aim to inform the public about Zika transmission routes, enhance monitoring and purification of water bodies, and promote the timely removal of stagnant water. Assume there exists ${\bar{u}}_{i}\in \left(0,1\right)$ such that $0\leq u_{i}\left(t\right)\leq {\bar{u}}_{i}<1$ for *i* = 1, 2, 3, 4. Within a fixed time interval [0, *T*] and for *t* > 0, the feasible decision space or constraint set is defined as follows:

(33) $$U=\left\{\left(u_{1}\left(t\right),u_{2}\left(t\right),u_{3}\left(t\right),u_{4}\left(t\right)\right)|0\leq u_{i}\left(t\right)\leq {\bar{u}}_{i},0\leq t\leq T,u_{i}\left(t\right)\text{is the Lebesgue measurable},i=1,2,3,4\right\},$$

In most studies on optimal control, the primary objective is often to minimize the number of infected individuals, thereby determining the optimal control strategy. However, such a goal may not adequately balance resource allocation across various aspects. Therefore, in this study, we adopt cost as the primary objective. In addition to minimizing the costs associated with releasing sterile mosquitoes and reducing human-to-mosquito transmission through various measures, we also aim to minimize the economic losses caused by the removal of adult and juvenile mosquito populations and the treatment of infected human cases. These costs are recorded in the interval [0, *T*] as follows:

(34) $$\begin{array}{ll} & \int_{0}^{T}\left(D_{1}\left(S_{H}\left(t\right)+S_{V}\left(t\right)\right)u_{1}\left(t\right)\right)dt,\int_{0}^{T}\left(D_{2}\left(S_{V}\left(t\right)+I_{V}\left(t\right)+G\left(t\right)\right)u_{2}\left(t\right)\right)dt,\\ & \int_{0}^{T}D_{3}N_{V}\left(t\right)u_{3}\left(t\right)dt,\int_{0}^{T}W_{1}S_{J}\left(t\right)u_{4}\left(t\right)dt,\int_{0}^{T}W_{2}I_{H}\left(t\right)dt, \end{array}$$

here, *D*_1_ is the cost of controlling human-mosquito transmission, *D*_2_ denotes the economic loss of killing mosquitoes with insecticides etc., *D*_3_ is the cost of releasing sterile mosquitoes, *W*_1_ is the cost of controlling environmental-mosquito transmission, and *W*_2_ is the cost of healthcare resources per case of ZIKV infection.

We consider the following multi-objective optimal problem

(35) $$\begin{array}{ll} & \min\int_{0}^{T}\left(D_{1}\left(S_{H}\left(t\right)+S_{V}\left(t\right)\right)u_{1}\left(t\right)\right)dt,\min\int_{0}^{T}\left(D_{2}\left(S_{V}\left(t\right)+I_{V}\left(t\right)+G\left(t\right)\right)u_{2}\left(t\right)\right)dt,\\ & \min\int_{0}^{T}D_{3}N_{V}\left(t\right)u_{3}\left(t\right)dt,\min\int_{0}^{T}W_{1}S_{J}\left(t\right)u_{4}\left(t\right)dt,\min\int_{0}^{T}W_{2}I_{H}\left(t\right)dt, \end{array}$$

The optimal control model is given by

(36) $$\left\{\begin{array}{ll} & \frac{dS_{H}}{dt}=\Lambda -r\beta_{H}\left(1-u_{1}\right)S_{H}I_{V}-\mu_{H}S_{H},\\ & \frac{dI_{H}}{dt}=r\beta_{H}\left(1-u_{1}\right)S_{H}I_{V}-\left(\mu_{H}+\delta +\eta \right)I_{H},\\ & \frac{dR_{H}}{dt}=\eta I_{H}-\mu_{H}R_{H},\\ & \frac{dS_{J}}{dt}=\frac{\sigma N_{V}}{N_{V}+G+\gamma }N_{V}-\alpha S_{J}-\beta \left(1-u_{4}\right)S_{J}V-\left(d_{0}+d_{1}\left(S_{J}+I_{J}\right)\right)S_{J},\\ & \frac{dI_{J}}{dt}=\beta \left(1-u_{4}\right)S_{J}V-\alpha I_{J}-\left(d_{0}+d_{1}\left(S_{J}+I_{J}\right)\right)I_{J},\\ & \frac{dS_{V}}{dt}=\alpha S_{J}-r\beta_{V}\left(1-u_{1}\right)S_{V}I_{H}-\mu_{1}\left(1+u_{2}\right)S_{V},\\ & \frac{dI_{V}}{dt}=\alpha I_{J}+r\beta_{V}\left(1-u_{1}\right)S_{V}I_{H}-\mu_{1}\left(1+u_{2}\right)I_{V},\\ & \frac{dV}{dt}=\theta I_{H}-\mu V,\\ & \frac{dG}{dt}=b\left(1-u_{3}\right)N_{V}-\mu_{2}\left(1+u_{2}\right)G. \end{array}\right)$$

The initial condition is

(37) $$\begin{array}{ll} & 0\leq S_{H}\left(0\right)\leq K,0\leq I_{H}\left(0\right)\leq K,0\leq R_{H}\left(0\right)\leq K,0\leq S_{J}\left(0\right)\leq K,0\leq I_{J}\left(0\right)\leq K,\\ & 0\leq S_{V}\left(0\right)\leq K,0\leq I_{V}\left(0\right)\leq K,0\leq V\left(0\right)\leq K,0\leq G\left(0\right)\leq K, \end{array}$$

where *K* is the positive constant.

In terms of methodology, there are several approaches to solving general sequential multi-objective optimization problems (Marler & Arora, 2004). One of the most common methods is the weighted sum or scalarization method. This method combines multiple objectives into a single scalar composite function, treating them either equally or with assigned weights, and minimizes the weighted convex sum of the objectives. Using the weighted sum method, we transform the vector-valued optimization problem (35)–(37) into a scalar quadratic optimization problem with a unique objective function of the form:

(38) $$\begin{array}{ll} & J\left(u_{1}\left(t\right),u_{2}\left(t\right),u_{3}\left(t\right),u_{4}\left(t\right)\right)=\int_{0}^{T}\left[\omega_{1}{\left(D_{1}\left(S_{H}\left(t\right)+S_{V}\left(t\right)\right)u_{1}\left(t\right)\right)}^{2}+\omega_{2}{\left(D_{2}\left(S_{V}\left(t\right)+I_{V}\left(t\right)+G\left(t\right)\right)u_{2}\left(t\right)\right)}^{2}\right)\\ & \left(+\omega_{3}{\left(D_{3}N_{V}\left(t\right)u_{3}\left(t\right)\right)}^{2}+\omega_{4}{\left(W_{1}S_{J}\left(t\right)u_{4}\left(t\right)\right)}^{2}+\omega_{5}{\left(W_{2}I_{H}\left(t\right)\right)}^{2}\right]dt, \end{array}$$

where Eq. (38) satisfies Eq. (36) and Eq. (37), and $\overset{5}{\underset{i=1}{\sum }}\omega_{i}=1$, *ω*_*i*_ ∈ [0, 1]. Here, the objective function

$$J\left(u_{1}\left(t\right),u_{2}\left(t\right),u_{3}\left(t\right),u_{4}\left(t\right)\right)$$

measures the total economic loss caused by the ZIKV.

To facilitate the following discussion, let *X* = (*S*_*H*_, *I*_*H*_, *R*_*H*_, *S*_*J*_, *I*_*J*_, *S*_*V*_, *I*_*V*_, *V*, *G*), *u* = (*u*_1_(*t*), *u*_2_(*t*), *u*_3_(*t*), *u*_4_(*t*)), $F\left(t,X,u\right)={\left(f_{1},f_{2},\ldots ,f_{9}\right)}^{\prime }$, where *f*_*i*_ denotes the *i*th right-hand side function in Eq. (35). Then the above single-objective optimal problem can be rewritten as

(39) $$\underset{\left(u_{1}\left(t\right),u_{2}\left(t\right),u_{3}\left(t\right),u_{4}\left(t\right)\right)\in U}{\min}J\left(u_{1}\left(t\right),u_{2}\left(t\right),u_{3}\left(t\right),u_{4}\left(t\right)\right),$$

(40) $$\frac{dX}{dt}=F\left(t,X,u\right),X\left(0\right)\geq 0,$$

where $\left\|X\left(0\right)\right\|\leq 3M$, and *ω*_*i*_ ∈ [0, 1] such that $\overset{5}{\underset{i=1}{\sum }}\omega_{i}=1$.

Theorem 8 *The optimal control of the optimization problem* (35) *is*

(41) $$\begin{array}{ll} & u_{1}^{*}\left(t\right)=min\left\{{\bar{u}}_{1},max\left\{u_{1},0\right\}\right\},u_{2}^{*}\left(t\right)=min\left\{{\bar{u}}_{2},max\left\{u_{2},0\right\}\right\},\\ & u_{3}^{*}\left(t\right)=min\left\{{\bar{u}}_{3},max\left\{u_{3},0\right\}\right\},u_{4}^{*}\left(t\right)=min\left\{{\bar{u}}_{4},max\left\{u_{4},0\right\}\right\}, \end{array}$$

*where*

$$\begin{array}{ll} & u_{1}=\frac{\left(\varphi_{2}-\varphi_{1}\right)r\beta_{H}I_{V}\left(t\right)S_{H}\left(t\right)+\left(\varphi_{7}-\varphi_{6}\right)r\beta_{V}S_{V}\left(t\right)I_{H}\left(t\right)}{2\omega_{1}D_{1}^{2}{\left(S_{H}\left(t\right)+S_{V}\left(t\right)\right)}^{2}},\\ & u_{2}=\frac{\mu_{1}\left(S_{V}\left(t\right)\varphi_{6}+I_{V}\left(t\right)\varphi_{7}\right)}{2\omega_{2}D_{2}^{2}{\left(S_{V}\left(t\right)+I_{V}\left(t\right)+G\left(t\right)\right)}^{2}},u_{3}=\frac{\varphi_{9}b}{2\omega_{3}D_{3}^{2}N_{V}\left(t\right)},u_{4}=\frac{\left(\varphi_{5}-\varphi_{4}\right)V}{2\omega_{4}W_{1}^{2}S_{J}\left(t\right)}. \end{array}$$

***Proof*.** *For ease of discussion, we note that* $X={\left(S_{H}\left(t\right),I_{H}\left(t\right),R_{H}\left(t\right),S_{J}\left(t\right),I_{J}\left(t\right),S_{V}\left(t\right),I_{V}\left(t\right),V\left(t\right),G\left(t\right)\right)}^{\prime }$ *corresponds to optimal control* $u^{*}={\left(u_{1}^{*}\left(t\right),u_{2}^{*}\left(t\right),u_{3}^{*}\left(t\right),u_{4}^{*}\left(t\right)\right)}^{\prime }$*. According to Pontryagin's Maximum Value Principle* (Flores et al., 2003; Andraud et al., 2012; Chitnis et al., 2008)*, the optimal control of* (39) − (40) *is equivalent to minimising the following Hamiltonian equation*

$$\begin{array}{ll} & H=\omega_{1}{\left(D_{1}\left(S_{H}\left(t\right)+S_{V}\left(t\right)\right)u_{1}\left(t\right)\right)}^{2}+\omega_{2}{\left(D_{2}\left(S_{V}\left(t\right)+I_{V}\left(t\right)+G\left(t\right)\right)u_{2}\left(t\right)\right)}^{2}\\ & +\omega_{3}{\left(D_{3}N_{V}\left(t\right)u_{3}\left(t\right)\right)}^{2}+\omega_{4}{\left(W_{1}S_{J}\left(t\right)u_{4}\left(t\right)\right)}^{2}+\omega_{5}{\left(W_{2}I_{H}\left(t\right)\right)}^{2}+\overset{9}{\underset{i=1}{\sum }}\varphi_{i}f_{i}, \end{array}$$

*where* *φ*_*i*_(*i* = 1, 2, …, 9) *are the concomitant variables that fulfil the following concomitant equations*

$$\begin{array}{ll} & \varphi_{1}^{\prime }=-\frac{\partial H}{\partial S_{H}}=-2\omega_{1}D_{1}^{2}u_{1}^{2}\left(t\right)\left(S_{H}\left(t\right)+S_{V}\left(t\right)\right)+\left(\varphi_{1}-\varphi_{2}\right)r\beta_{H}\left(1-u_{1}\right)I_{V}+\varphi_{1}\mu_{H},\\ & \varphi_{2}^{\prime }=-\frac{\partial H}{\partial I_{H}}=-2\omega_{5}W_{2}^{2}I_{H}\left(t\right)+\varphi_{2}\left(\mu_{H}+\delta \right)+\left(\varphi_{2}-\varphi_{3}\right)\eta +\left(\varphi_{6}-\varphi_{7}\right)r\beta_{V}\left(1-u_{1}\right)S_{V}-\varphi_{8}\theta ,\\ & \varphi_{3}^{\prime }=-\frac{\partial H}{\partial R_{H}}=\varphi_{3}\mu_{H},\varphi_{4}^{\prime }=-\frac{\partial H}{\partial S_{J}}=-2\omega_{4}W_{1}^{2}u_{4}^{2}\left(t\right)S_{J}\left(t\right)+\left(\varphi_{4}-\varphi_{6}\right)\alpha \\ & +\left(\varphi_{4}-\varphi_{5}\right)\beta \left(1-u_{4}\right)V+\varphi_{4}\left(d_{0}+\left(2S_{J}+I_{J}\right)d_{1}\right)+\varphi_{5}d_{1}I_{J},\\ & \varphi_{5}^{\prime }=-\frac{\partial H}{\partial I_{J}}=\varphi_{4}d_{1}S_{J}+\left(\varphi_{5}-\varphi_{7}\right)\alpha +\varphi_{5}\left(d_{0}+\left(2I_{J}+S_{J}\right)d_{1}\right),\\ & \varphi_{6}^{\prime }=-\frac{\partial H}{\partial S_{V}}=-2\omega_{1}D_{1}^{2}u_{1}^{2}\left(t\right)\left(S_{H}\left(t\right)+S_{V}\left(t\right)\right)-2\left(S_{V}\left(t\right)+I_{V}\left(t\right)\right)\left(\omega_{2}D_{2}^{2}u_{2}^{2}\left(t\right)-\omega_{3}D_{3}^{2}u_{3}^{2}\left(t\right)\right)\\ & -\varphi_{4}\frac{\sigma N_{V}\left[N_{V}+2\left(G+\gamma \right)\right]}{{\left(N_{V}+G+\gamma \right)}^{2}}+\left(\varphi_{6}-\varphi_{7}\right)r\beta_{V}\left(1-u_{1}\right)I_{H}+\varphi_{7}\left(1+u_{2}\right)\mu_{1}-\varphi_{9}b\left(1-u_{3}\right), \end{array}$$

$$\begin{array}{ll} & \varphi_{7}^{\prime }=-\frac{\partial H}{\partial I_{V}}=-2\omega_{2}D_{2}^{2}u_{2}^{2}\left(t\right)\left(S_{V}\left(t\right)+I_{V}\left(t\right)\right)-2\omega_{3}D_{3}^{2}u_{3}^{2}\left(t\right)\left(S_{V}\left(t\right)+I_{V}\left(t\right)\right)\\ & +\left(\varphi_{1}-\varphi_{2}\right)r\beta_{H}\left(1-u_{1}\right)S_{H}-\varphi_{4}\frac{\sigma N_{V}\left[N_{V}+2\left(G+\gamma \right)\right]}{{\left(N_{V}+G+\gamma \right)}^{2}}+\varphi_{7}\left(1+u_{2}\right)\mu_{1}-\varphi_{9}b\left(1-u_{3}\right),\\ & \varphi_{8}^{\prime }=-\frac{\partial H}{\partial V}=\left(\varphi_{4}-\varphi_{5}\right)\beta \left(1-u_{4}\right)S_{J}+\varphi_{8}\mu ,\\ & \varphi_{9}^{\prime }=-\frac{\partial H}{\partial G}=-2\omega_{2}D_{2}^{2}u_{2}^{2}\left(t\right)G\left(t\right)+\varphi_{4}\frac{\sigma N_{V}^{2}}{{\left(N_{V}+G+\gamma \right)}^{2}}+\varphi_{9}\mu_{2}. \end{array}$$

*Concurrent horizontal conditions*

(42) $$\varphi_{i}\left(T\right)=0\left(i=1,2,\ldots ,9\right),$$

*hence we have*

$$\begin{array}{ll} & \frac{\partial H}{\partial u_{1}}=2\omega_{1}D_{1}^{2}{\left(S_{H}\left(t\right)+S_{V}\left(t\right)\right)}^{2}u_{1}\left(t\right)+\left(\varphi_{1}-\varphi_{2}\right)r\beta_{H}I_{V}S_{H}+\left(\varphi_{6}-\varphi_{7}\right)r\beta_{V}S_{V}I_{H}=0,\\ & \frac{\partial H}{\partial u_{2}}=2\omega_{2}D_{2}^{2}{\left(S_{V}\left(t\right)+I_{V}\left(t\right)+G\left(t\right)\right)}^{2}u_{2}\left(t\right)-\mu_{1}\left(S_{V}\varphi_{6}+I_{V}\varphi_{7}\right)=0,\\ & \frac{\partial H}{\partial u_{3}}=2\omega_{3}D_{3}^{2}N_{V}^{2}\left(t\right)u_{3}\left(t\right)-\varphi_{9}bN_{V}=0,\frac{\partial H}{\partial u_{4}}=2\omega_{4}W_{1}^{2}S_{J}^{2}\left(t\right)u_{4}\left(t\right)+\left(\varphi_{4}-\varphi_{5}\right)\beta S_{J}V=0. \end{array}$$

Solving for this gives

$$\begin{array}{ll} & u_{1}=\frac{\left(\varphi_{2}-\varphi_{1}\right)r\beta_{H}I_{V}S_{H}+\left(\varphi_{7}-\varphi_{6}\right)r\beta_{V}S_{V}I_{H}}{2\omega_{1}D_{1}^{2}{\left(S_{H}\left(t\right)+S_{V}\left(t\right)\right)}^{2}},\\ & u_{2}=\frac{\mu_{1}\left(S_{V}\varphi_{6}+I_{V}\varphi_{7}\right)}{2\omega_{2}D_{2}^{2}{\left(S_{V}\left(t\right)+I_{V}\left(t\right)+G\left(t\right)\right)}^{2}},u_{3}=\frac{\varphi_{9}b}{2\omega_{3}D_{3}^{2}N_{V}\left(t\right)},u_{4}=\frac{\left(\varphi_{5}-\varphi_{4}\right)V}{2\omega_{4}W_{1}^{2}S_{J}\left(t\right)}. \end{array}$$

*Moreover, in view of* $\left(u_{1}^{*}\left(t\right),u_{2}^{*}\left(t\right),u_{3}^{*}\left(t\right),u_{4}^{*}\left(t\right)\right)\in U$*, i.e., using the upper and lower bounds of* *u*∗*, we find that the optimal control* $u^{*}=\left(u_{1}^{*}\left(t\right),u_{2}^{*}\left(t\right),u_{3}^{*}\left(t\right),u_{4}^{*}\left(t\right)\right)$ *can be well expressed by* Eq. (41) *is well expressed.*

## 6 Numerical simulations

In this section, numerical simulations are carried out to verify the above theoretical analysis, and the values of some fixed parameters are listed in Table 2.

**Table 2:** Values of fixed parameters in model (2).

Parameter	Range	Value	References
*μ*_*H*_	(3.6 × 10^−5^, 6.0 × 10^−5^) per day	0.00005	Hui et al., 2023Hui et al. (2023)
*δ*	(0, 4.1 × 10^−4^) per day	0.00035	Wan et al., 2009Wan et al. (2009)
*η*	(0, 1) per day	0.5	Assumed
*r*	(0.3, 1) per day	0.3	Andraud et al., 2012Andraud et al. (2012)
*β*_*H*_	(0, 1) per day	0.2	Lambrechts et al., 2011Lambrechts et al. (2011)
*β*_*V*_	(0, 0.97) per day	0.01	Lambrechts et al., 2011Lambrechts et al. (2011)
*α*	(0, 1) per day	0.5	Assumed
*σ*	(0.9043, 6.4594) per unit of time	1	Hui et al., 2023Hui et al. (2023)
*θ*	(0, 1) per day	0.1	Wang et al., 2021Wang et al. (2021)
*μ*	(0, 1) per day	0.1	Wang et al., 2021Wang et al. (2021)
*μ*_1_	(0, 1) per day	0.2	Assumed
*μ*_2_	(0, 1) per day	0.22	Assumed
*γ*	1	1	Assumed

### 6.1 Dynamical behaviour of models considering the diffusion of disease (11)

**Fig. 2:**
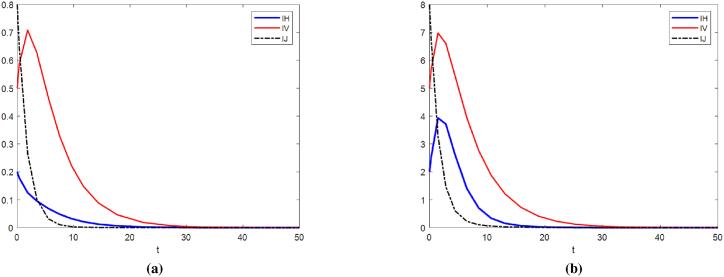
Time series plot of model (11) at different initial values when *R*_0_ < 1. (a)Initial values is [1, 0.2, 0.5, 0.8, 0.3, 1, 2, 1]; (b)Initial values is [10, 2, 5, 8, 3, 6, 2, 5].

We set Λ = 0.0001, *d*_0_ = 0.1, *d*_1_ = 0.001, *b* = 0.6, *β* = 0.01, we can get the basic reproduction number *R*_0_ = 0.9631 < 1. According to Theorem 4, the disease-free equilibrium of model (11) *E*_0_ is globally asymptotically stable. It can be seen from Fig. 2 that *I*_*H*_, *I*_*V*_ and *I*_*J*_ eventually converge to 0 no matter which set of initial values is taken.

**Fig. 3:**
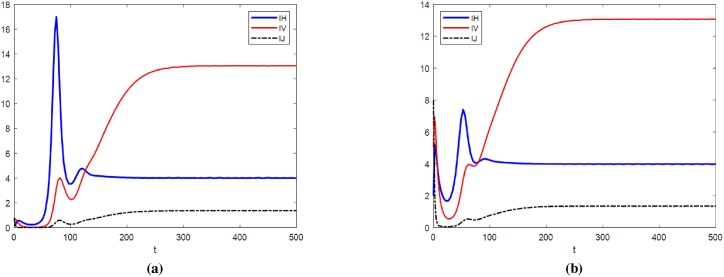
Time series plot of model (11) at different initial values when *R*_0_ > 1. (a) Initial values is [1, 0.2, 0.5, 0.8, 0.3, 1, 2, 1]; (b) Initial values is [10, 2, 5, 8, 3, 6, 2, 5].

Assuming Λ = 2, *d*_0_ = 0.1, *d*_1_ = 0.001, *b* = 0.6, *β* = 0.01, the reproduction regeneration number *R*_0_ = 129.23 > 1. At this point, a stable endemic equilibrium *E*∗ exists (Fig. 3).

### 6.2 Effect of release ratio in sterile mosquitoes on ZIKV transmission

The release of a certain number of sterile mosquitoes can reduce the population of wild mosquitoes, thereby lowering the risk of mosquito-borne disease transmission. In our model, the release ratio of sterile mosquitoes, denoted as *b*, represents the number of sterile mosquitoes released. In this subsection, we will further investigate the impact of the release ratio *b* on ZIKV transmission.

**Fig. 4:**
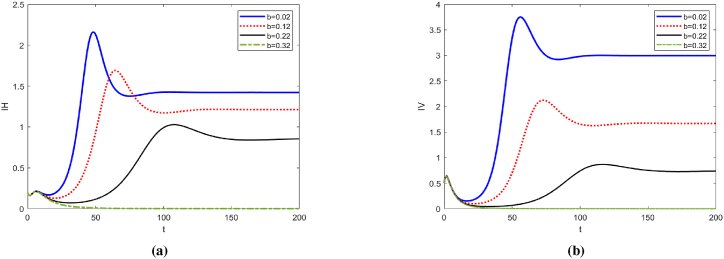
Trend plots of *I*_*H*_ and *I*_*V*_ under different release ratios.

We set Λ = 1, *d*_0_ = 0.3, *d*_1_ = 0.05, *β* = 0.1, *μ*_*H*_ = 0.05, the initial value of [1, 0.2, 0.5, 0.8, 0.3, 1, 2, 1]. Under these parameter conditions, we calculated *b*_*p*_ = 0.4658 and obtained *b*_*p*_ = 0.4658. Considering the case of non-extinction of mosquito population, the release rates of sterile mosquitoes were taken as 0.02, 0.12, 0.22 and 0.32. From Fig. 4, we can observe that as the release coefficient *b* increases, the number of infected individuals and infected mosquitoes decreases significantly. Additionally, the peak time of infection is delayed, and the peak value is also notably reduced.

**Fig. 5:**
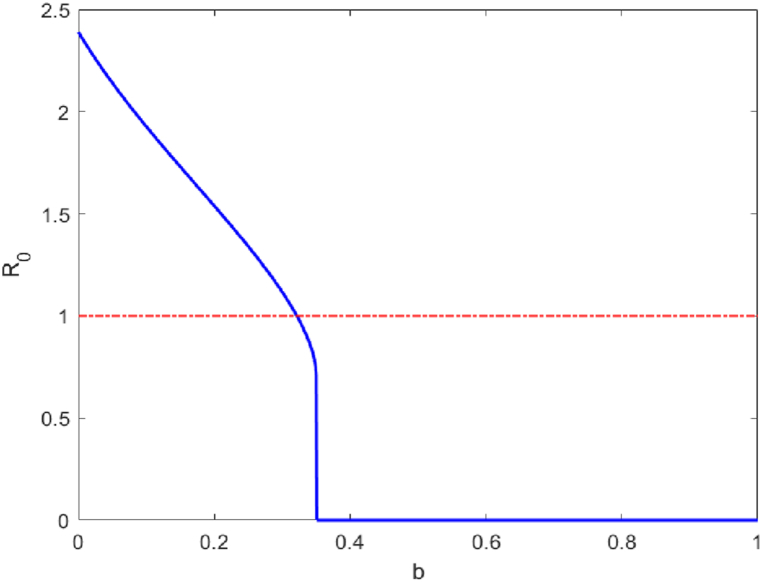
Correlation between release ratio in sterile mosquitoes *b* and basic regeneration number *R*_0_.

Fig. 5 illustrates the relationship between the basic reproduction number *R*_0_ and the release ratio of sterile mosquitoes *b*. As *b* increases, *R*_0_ decreases, indicating that the risk of ZIKV transmission also decreases with a higher release ratio of sterile mosquitoes. When *b* > 0.32, *R*_0_ will be less than 1, and the spread of the disease can be effectively controlled.

### 6.3 Impact of contaminated aquatic environments on ZIKV transmission

ZIKV is mainly distributed by *Aedes aegypti* mosquitoes, and the breeding sites of these mosquitoes are usually associated with water bodies. Pollution of aquatic environments provides breeding sites for mosquitoes and promotes the growth and reproduction of mosquito larvae. Therefore, in this subsection, we explore how contaminated aquatic environments affect ZIKV transmission.

**Fig. 6:**
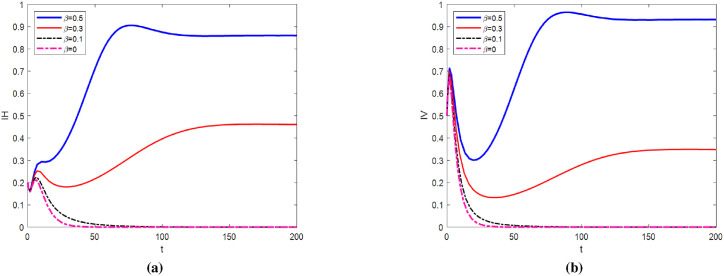
Variation trends of *I*_*H*_ and *I*_*V*_ with *β*.

Take Λ = 1, *d*_0_ = 0.1, *d*_1_ = 0.1, *b* = 0.4, *μ*_*H*_ = 0.06. From Fig. 6, it can be observed that as the probability *β* of mosquitoes acquiring the virus from the environment increases from 0 to 0.5, the number of infected individuals and mosquitoes rises significantly. Even though the release rate of sterile mosquitoes reaches 0.4, the number of infections still increases when the environmental infection probability is relatively high (e.g., *β* = 0.3). This reflects the significant role of the environmental transmission pathway in promoting virus spread. The probability of environmental infection is closely linked to the concentration of the virus in the environment. However, environmental control measures and their intensity are often overlooked, which may create opportunities for the virus to resurge.

### 6.4 Optimal control results and discussion

In this subsection, using the Forward-Backward sweep methods (Lenhart & Workman, 2007), we elaborate the optimal control further through numerical experiments. According to the Global Response to Vector Control 2017–2030, the average annual per capital cost of mosquito nets is 1.27 (*D*_1_ = 1.27), the yearly per capita cost of insecticides is about 4.24 (*D*_2_ = 4.24), the yearly per capita cost of healthcare resources for ZIKA virus cases is 3 (*W*_2_ = 3), and the cost of health promotion and education per person per year is more than 1, we take 1.1 (*W*_1_ = 1.1) per person per year. According to Guo et al. (2022), sterile male mosquitoes were released by drones. The number of males released is strictly limited to 50,000 at a time, and the cost per hectare per week is only 1. We estimated the cost of mass-producing sterile mosquitoes to be 0.5 per mosquito, and calculated that the cost of releasing each sterile male per hectare per year would be approximately 0.0096 (*D*_3_ = 0.0096).

We take Λ = 1, *β*_*V*_ = 0.09, *b* = 0.6, *θ* = 0.3, *β* = 0.05, and the other parameters are shown in Table 2. The three weighting ratio configurations are defined as:

(1) Balanced regulation strategy(*ω*_1_ = *ω*_2_ = *ω*_3_ = *ω*_4_ = *ω*_5_ = 0.2);
(2) Prioritized control of insecticide expenditure and healthcare costs(*ω*_1_ = 0.2, *ω*_2_ = *ω*_5_ = 0.3, *ω*_3_ = *ω*_4_ = 0.1);
(3) Prioritized control of sterile insect release and environmental cleaning costs(*ω*_1_ = *ω*_2_ = 0.1, *ω*_3_ = *ω*_4_ = 0.3, *ω*_5_ = 0.2).

**Fig. 7:**
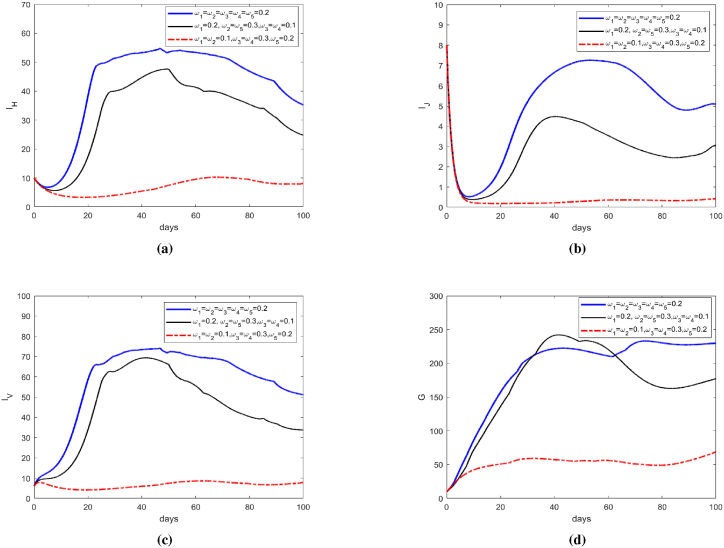
Optimal Control of *I*_*H*_, *I*_*J*_, *I*_*V*_ and *G*.

Fig. 7 simulates the trends of the number of infected humans (*S*_*H*_), the number of infected larval mosquitoes (*I*_*J*_), the number of infected adult mosquitoes (*I*_*V*_), and the number of infertile mosquitoes (*G*) under three different sets of weight values. As illustrated in Fig. 7, Configuration 3 demonstrates superior epidemic containment by maintaining infections at relatively low levels throughout the intervention period. Compared to the balanced control strategy (Configuration 1), this scheme achieves a 78 % reduction in cumulative infection scale. Notably, the diminished population of infected mosquitoes proportionally reduces required sterile insect release volumes.

**Fig. 8:**
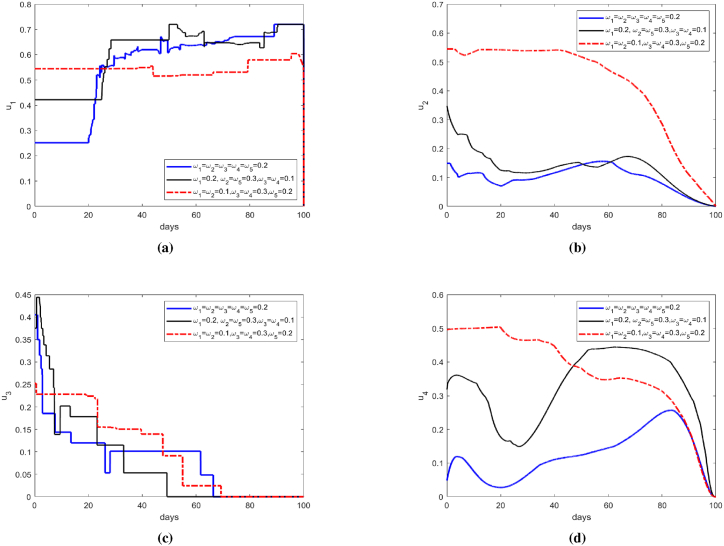
Optimal control of *u*_1_, *u*_2_, *u*_3_ and *u*_4_.

The dynamic progression shown in Fig. 8 reveals four characteristic phases under Configuration 3:

(1) Protective measures (e.g., bed nets) require sustained high-intensity implementation (55–60 % coverage).
(2) Insecticide application displays gradual attenuation from Day 40 onward.
(3) The release intensity of sterile mosquitoes increases progressively, reaching about 95 % of the predetermined maximum release ratio by around the 55th day.
(4) Environmental management inputs maintain slow reduction trajectories from initial moderate levels.

This phased implementation framework ensures optimal resource allocation while achieving predefined epidemic control targets. The observed 15 day delay between sterile release initiation and measurable population suppression aligns with established mosquito reproductive cycles. Of course, from the control comparison, it can be clearly seen that during a virus outbreak, the short-term effect of using insecticides is significantly higher than the release of sterile mosquitoes. Therefore, the release control of sterile mosquitoes is more suitable for daily protective work to reduce the risk of virus outbreaks.

## 7 Discussion and conclusions

The application of SIT in mosquito-borne disease control has been a significant trend. In this paper, we developed an innovative ZIKV transmission control model based on SIT, with special emphasis on the mosquito vector transmission process and the possible transmission routes of mosquitoes carrying the virus in aquatic environments. Similar to traditional control methods, this paper aims to reduce the mosquito population through the release of sterile insects to control the transmission of the virus effectively rather than eliminating the mosquito population completely. Therefore, the study focuses on the coexistence of sterile mosquitoes and wild mosquitoes and its effect on virus transmission.

Through dynamic behavioural analysis, we found that there exists a critical threshold *b*_*p*_ for the release of sterile insects. When the release rate *b* is lower than *b*_*p*_ and the Allee effect is insignificant (*r* < *r*_*p*_), the system will reach a stable coexistence state of the two mosquitoes; otherwise, both mosquitoes will become extinct. Although Fig. 1 shows that low densities of wild mosquitoes may also lead to population extinction, from the perspective of controlling the spread of infectious diseases, low densities of wild mosquitoes do not cause large-scale transmission of viruses. However, when a virus outbreak occurs, the population density of wild mosquitoes must reach a certain size in order to cause a rapid increase in the number of infected people. Therefore, this study identifies the key node values *b*_*p*_ and *r*_*p*_ for controlling the transmission of ZIKV using the sterile insect technique and reveals the intrinsic relationship between them.

Further, under the condition of stable co-existence of released sterile mosquitoes and wild mosquitoes, we give the basic reproduction number *R*_0_ for ZIKV transmission, analyse the stability of disease-free equilibrium and endemic equilibrium, and prove that the system is consistently persistent when *R*_0_ > 1, which implies that the virus will continue to spread. The result provides theoretical support for the long-term dynamics of virus propagation.

In addition, we propose a comprehensive optimal control strategy under cost constraints. Unlike conventional optimal control models that focus solely on minimizing the number of infected individuals, we have developed a multi-objective optimization model aimed at minimizing control costs while analyzing optimal strategies under varying weight allocations. The results demonstrate that during viral outbreaks, the short-term efficacy of insecticides remains critical. However, over time, the impact of sterile mosquito releases becomes pronounced, allowing insecticide usage to be phased out and biological control to progressively replace chemical interventions. Given the latency period associated with sterile mosquito deployment, integrating such releases during routine viral prevention phases can significantly mitigate outbreak risks. This strategy offers a novel perspective for managing disease transmission in resource-limited settings. This paper also focuses on the environmental infection pathway of ZIKV. Although this pathway accelerates the accumulation of infected individuals, the introduction of sterile insect technology significantly reduces the risk. The results of this study not only provide a scientific basis for the control of ZIKV, but also provide theoretical support for the control of other mosquito-borne diseases such as dengue fever and malaria.

## CRediT authorship contribution statement

**Zongmin Yue:** Writing – review & editing, Supervision, Methodology, Funding acquisition, Conceptualization. **Yingpan Zhang:** Writing – original draft, Software, Investigation, Formal analysis. **Xiangrui Ji:** Software.

## Use of AI tools declaration

The authors declare they have not used Artificial Intelligence (AI) tools in the creation of this article.

## Declaration of competing interest

The authors declare that they have no competing interests.

## Appendix A

The Jacobi matrix of model (3) at the zero equilibrium point ${\bar{E}}_{0}$ is

$$J_{0}\left({\bar{E}}_{0}\right)=\left(\begin{array}{lll} -\left(\alpha +d_{0}\right) & 0 & 0\\ \alpha & -\mu_{1} & 0\\ 0 & b & -\mu_{2} \end{array}\right),$$

Since the eigenvalues of $J\left({\bar{E}}_{0}\right)$ are −(*α* + *d*_0_) < 0, − *μ*_1_ < 0, − *μ*_2_ < 0, ${\bar{E}}_{0}$ is locally asymptotically stable. In particular, when *r*_*p*_ > 1 and *b* ≥ *b*_*p*_, ${\bar{E}}_{0}=\left(0,0,0\right)$ is the unique equilibrium point for model (3), combined with the boundedness of the modelled solution, it can be seen that ${\bar{E}}_{0}$ is globally asymptotically stable; when *r*_*p*_ > 1, *b* < *b*_*p*_ and Δ_*p*_ < 0, ${\bar{E}}_{0}$ is also globally asymptotically stable.

Here, the stability of the positive equilibrium point is discussed. For *r*_*p*_ > 1,*b* < *b*_*p*_ and Δ_*p*_ ≥ 0, the Jacobi matrix of model (3) at the positive equilibrium point ${\bar{E}}^{*}$ is

$$J_{1}\left({\bar{E}}^{*}\right)=\left(\begin{array}{lll} -\left(\alpha +d_{0}+2d_{1}J\right) & \frac{\sigma N_{V}}{N_{V}+G+\gamma }\left(1+\frac{G+\gamma }{N_{V}+G+\gamma }\right) & -\frac{\sigma N_{V}^{2}}{{\left(N_{V}+G+\gamma \right)}^{2}}\\ \alpha & -\mu_{1} & 0\\ 0 & b & -\mu_{2} \end{array}\right).$$

Let

$$\xi_{11}=\alpha +d_{0}+2d_{1}J,\xi_{12}=\frac{\sigma N_{V}}{N_{V}+G+\gamma }\left(1+\frac{G+\gamma }{N_{V}+G+\gamma }\right),\xi_{13}=\frac{\sigma N_{V}^{2}}{{\left(N_{V}+G+\gamma \right)}^{2}},$$

then

$$\left|\begin{array}{l} \lambda E-J_{1}\left({\bar{E}}^{*}\right) \end{array}\right|=\left|\begin{array}{lll} \lambda +\xi_{11} & -\xi_{12} & \xi_{13}\\ -\alpha & \lambda +\mu_{1} & 0\\ 0 & -b & \lambda +\mu_{2} \end{array}\right|=0,$$

The eigenvalues of $J_{1}\left({\bar{E}}^{*}\right)$ content the following equation:

$$\lambda^{3}+B_{1}\lambda^{2}+B_{2}\lambda +B_{3}=0,$$

where

$$\begin{array}{ll} & B_{1}=\xi_{11}+\mu_{1}+\mu_{2}=\alpha +d_{0}+2d_{1}J+\mu_{1}+\mu_{2}>0,\\ & B_{2}=\mu_{1}\mu_{2}+\xi_{11}\left(\mu_{1}+\mu_{2}\right)-\alpha \xi_{12},\\ & B_{3}=\xi_{11}\mu_{1}\mu_{2}+\alpha b\xi_{13}-\alpha \mu_{2}\xi_{12}\\ & =\mu_{1}\mu_{2}\left(\alpha +d_{0}+2d_{1}J\right)-\frac{\mu_{2}^{2}\sigma \alpha^{2}}{\left[\left(\mu_{2}\alpha +\alpha b\right)J+\mu_{1}\mu_{2}\gamma \right]}-\frac{\mu_{1}\mu_{2}^{3}\gamma \sigma \alpha^{2}}{{\left[\left(\mu_{2}\alpha +\alpha b\right)J+\mu_{1}\mu_{2}\gamma \right]}^{2}}, \end{array}$$

therefore

$$B_{3}=\frac{\mu_{2}J}{\left(\left(\mu_{2}\alpha +\alpha b\right)J+\mu_{1}\mu_{2}\gamma \right)}N^{\prime }\left(J\right)-\frac{\mu_{2}\left(\mu_{2}\alpha +\alpha b\right)J}{{\left(\left(\mu_{2}\alpha +\alpha b\right)J+\mu_{1}\mu_{2}\gamma \right)}^{2}}N\left(J\right).$$

Due to $N\left(J_{1}^{*}\right)=0$, $N\left(J_{1}^{*}\right)<0$, $N\left(J_{2}^{*}\right)=0$, $N\left(J_{2}^{*}\right)>0$, so $B_{3}\left({\bar{E}}_{1}^{*}\right)<0$, $B_{3}\left({\bar{E}}_{2}^{*}\right)>0$, i.e. there is $\det\left(J_{1}\left({\bar{E}}_{1}^{*}\right)\right)>0$, $\det\left(J_{1}\left({\bar{E}}_{2}^{*}\right)\right)<0$. Therefore, ${\bar{E}}_{1}^{*}$ is an unstable saddle point. Next computing *B*_1_*B*_2_ − *B*_3_, we have

$$\begin{array}{ll} & B_{1}B_{2}-B_{3}=\left(\xi_{11}+\left(\mu_{1}+\mu_{2}\right)\right)\left(\mu_{1}\mu_{2}+\xi_{11}\left(\mu_{1}+\mu_{2}\right)-\alpha \xi_{12}\right)-\xi_{11}\mu_{1}\mu_{2}-\alpha b\xi_{13}+\alpha \mu_{2}\xi_{12}\\ & =\mu_{1}\mu_{2}\left(\mu_{1}+\mu_{2}\right)+{\left(\mu_{1}+\mu_{2}\right)}^{2}\xi_{11}+\xi_{11}\left(\xi_{11}\left(\mu_{1}+\mu_{2}\right)-\alpha \xi_{12}\right)-\alpha b\xi_{13}-\alpha \mu_{1}\xi_{12}. \end{array}$$

Take ${\hat{\xi }}_{11}=\frac{1}{J}\frac{\sigma N_{V}^{2}}{N_{v}+G+\gamma },{\bar{\xi }}_{11}=d_{1}J$, then

$$\xi_{11}=\alpha +d_{0}+2d_{1}J=\frac{1}{J}\left(\frac{\sigma N_{V}^{2}}{\left(N_{V}+G+\gamma \right)}+d_{1}J^{2}\right)={\hat{\xi }}_{11}+{\bar{\xi }}_{11},$$

$$\begin{array}{ll} & B_{1}B_{2}-B_{3}=\mu_{1}\mu_{2}\left(\mu_{1}+\mu_{2}\right)+{\left(\mu_{1}+\mu_{2}\right)}^{2}\xi_{11}+\xi_{11}\left(\xi_{11}\left(\mu_{1}+\mu_{2}\right)-\alpha \xi_{12}\right)-\alpha b\xi_{13}-\alpha \mu_{1}\xi_{12}\\ & =\mu_{1}\mu_{2}\left(\mu_{1}+\mu_{2}\right)+{\left(\mu_{1}+\mu_{2}\right)}^{2}\left({\hat{\xi }}_{11}+{\bar{\xi }}_{11}\right)-\alpha \mu_{1}\xi_{12}+\xi_{11}\left(\xi_{11}\left(\mu_{1}+\mu_{2}\right)-\alpha \xi_{12}\right)-\alpha b\xi_{13}\\ & =\mu_{1}\mu_{2}\left(\mu_{1}+\mu_{2}\right)+{\left(\mu_{1}+\mu_{2}\right)}^{2}{\bar{\xi }}_{11}+\mu_{1}\left(\left(\mu_{1}+\mu_{2}\right){\hat{\xi }}_{11}-\alpha \xi_{12}\right)+\mu_{2}\left(\mu_{1}+\mu_{2}\right){\hat{\xi }}_{11}-\alpha b\xi_{13}\\ & \left(+\xi_{11}\left(\xi_{11}\mu_{1}+\mu_{2}\right)-\alpha \xi_{12}\right)=\mu_{1}\mu_{2}\left(\mu_{1}+\mu_{2}\right)+{\left(\mu_{1}+\mu_{2}\right)}^{2}{\bar{\xi }}_{11}+B_{4}+\xi_{11}B_{5}. \end{array}$$

where,

$$\begin{array}{ll} & B_{4}=\mu_{1}\left(\left(\mu_{1}+\mu_{2}\right){\hat{\xi }}_{11}-\alpha \xi_{12}\right)+\mu_{2}\left(\mu_{1}+\mu_{2}\right){\hat{\xi }}_{11}-\alpha b\xi_{13}\\ & =\frac{\mu_{1}\left(\mu_{1}+\mu_{2}\right)\sigma N_{V}^{2}}{J\left(N_{V}+G+\gamma \right)}-\frac{\mu_{1}\alpha \sigma N_{V}}{N_{V}+G+\gamma }\left(1+\frac{G+\gamma }{N_{V}+G+\gamma }\right)+\frac{\mu_{2}\left(\mu_{1}+\mu_{2}\right)\sigma N_{V}^{2}}{J\left(N_{V}+G+\gamma \right)}-\frac{\mu_{1}\mu_{2}\sigma N_{V}^{2}G}{J{\left(N_{V}+G+\gamma \right)}^{2}}\\ & =\frac{\mu_{1}\left(\mu_{1}+\mu_{2}\right)\sigma N_{V}^{2}}{J\left(N_{V}+G+\gamma \right)}-\frac{\mu_{1}^{2}\sigma N_{V}^{2}}{J\left(N_{V}+G+\gamma \right)}\left(1+\frac{G+\gamma }{N_{V}+G+\gamma }\right)+\frac{\mu_{2}\left(\mu_{1}+\mu_{2}\right)\sigma N_{V}^{2}}{J\left(N_{V}+G+\gamma \right)}-\frac{\mu_{1}\mu_{2}\sigma N_{V}^{2}G}{J{\left(N_{V}+G+\gamma \right)}^{2}}\\ & =\frac{\mu_{1}\mu_{2}\sigma N_{V}^{2}}{J\left(N_{V}+G+\gamma \right)}-\frac{\mu_{1}^{2}\sigma N_{V}^{2}\left(G+\gamma \right)}{J{\left(N_{V}+G+\gamma \right)}^{2}}+\frac{\mu_{2}\left(\mu_{1}+\mu_{2}\right)\sigma N_{V}^{2}}{J\left(N_{V}+G+\gamma \right)}-\frac{\mu_{1}\mu_{2}\sigma N_{V}^{2}G}{J{\left(N_{V}+G+\gamma \right)}^{2}}\\ & =\frac{\mu_{1}\mu_{2}\sigma N_{V}^{2}}{J\left(N_{V}+G+\gamma \right)}\left(1-\frac{G}{N_{V}+G+\gamma }\right)+\frac{\sigma N_{V}^{2}}{J\left(N_{V}+G+\gamma \right)}\left(\mu_{2}^{2}+\mu_{1}\left(\mu_{2}-\mu_{1}\frac{G+\gamma }{N_{V}+G+\gamma }\right)\right), \end{array}$$

$$\begin{array}{ll} & B_{5}=\xi_{11}\left(\mu_{1}+\mu_{2}\right)-\alpha \xi_{12}=\left(\mu_{1}+\mu_{2}\right){\hat{\xi }}_{11}+\left(\mu_{1}+\mu_{2}\right){\bar{\xi }}_{11}-\alpha \xi_{12}\\ & =\frac{\sigma N_{V}^{2}\left(\mu_{1}+\mu_{2}\right)}{J\left(N_{V}+G+\gamma \right)}-\frac{\alpha \sigma N_{V}}{N_{V}+G+\gamma }\left(1+\frac{G+\gamma }{N_{V}+G+\gamma }\right)+\left(\mu_{1}+\mu_{2}\right){\bar{\xi }}_{11}\\ & =\frac{\sigma N_{V}^{2}\left(\mu_{1}+\mu_{2}\right)}{J\left(N_{V}+G+\gamma \right)}-\frac{\mu_{1}\sigma N_{V}^{2}}{J\left(N_{V}+G+\gamma \right)}\left(1+\frac{G+\gamma }{N_{V}+G+\gamma }\right)+\left(\mu_{1}+\mu_{2}\right){\bar{\xi }}_{11}\\ & =\frac{\sigma N_{V}^{2}}{J\left(N_{V}+G+\gamma \right)}\left(\mu_{2}-\mu_{1}\frac{G+\gamma }{N_{V}+G+\gamma }\right), \end{array}$$

Then when *μ*_2_ ≥ *μ*_1_, *B*_4_ > 0, *B*_5_ > 0, and thus *B*_1_*B*_2_ − *B*_3_ > 0. By the Hurwitz criterion, the positive equilibrium point ${\bar{E}}_{2}^{*}$ is locally asymptotically stable.

For *r*_*p*_ > 1, *b* < *b*_*p*_ and Δ_*p*_ = 0, we have: $\det\left(J_{1}\left({\bar{E}}_{0}^{*}\right)\right)=0$, so the positive equilibrium point ${\bar{E}}_{0}^{*}$ is locally asymptotically stable. We thus prove Theorem 1.

## Appendix B

The Jacobi matrix of model (7) at the zero equilibrium point ${\tilde{E}}_{0}$ is

$$J_{0}\left({\tilde{E}}_{0}\right)=\left(\begin{array}{ll} -\left(\alpha +d_{0}\right) & \sigma \\ \alpha & -\mu_{1} \end{array}\right),$$

the characteristic equation is *λ*^2^ + (*α* + *d*_0_ + *μ*_1_)*λ* + (*α* + *d*_0_)*μ*_1_ − *σα* = 0, thus we have

$$\begin{array}{ll} & \lambda_{1}+\lambda_{2}=-\left(\alpha +d_{0}+\mu_{1}\right)<0,\\ & \lambda_{1}\lambda_{2}=\left(\alpha +d_{0}\right)\mu_{1}-\sigma \alpha =\mu_{1}\left(\alpha +d_{0}\right)\left(1-\tilde{r_{p}}\right). \end{array}$$

When $\tilde{r_{p}}\geq 1$, *λ*_1_*λ*_2_ < 0. Therefore, for model (7), the zero equilibrium point ${\tilde{E}}_{0}$ is unstable.

The Jacobi matrix of model (7) at the positive equilibrium point ${\tilde{E}}_{0}^{*}$ is

$$J_{1}\left({\tilde{E}}_{0}^{*}\right)=\left(\begin{array}{ll} -\left(\alpha +d_{0}+2d_{1}J\right) & \sigma \\ \alpha & -\mu_{1} \end{array}\right),$$

so we obtain the following characteristic equation

(B1) $$\lambda^{2}+\left(\alpha +d_{0}+2d_{1}J+\mu_{1}\right)\lambda +\mu_{1}\left(\alpha +d_{0}+2d_{1}J\right)-\sigma \alpha =0.$$

Therefore, we have

$$\begin{array}{ll} & \lambda_{1}+\lambda_{2}=-\left(\alpha +d_{0}+2d_{1}J+\mu_{1}\right)<0,\\ & \lambda_{1}\lambda_{2}=\mu_{1}\left(\alpha +d_{0}+2d_{1}J\right)-\sigma \alpha =\sigma \alpha -\mu_{1}\left(\alpha +d_{0}\right)=\mu_{1}\left(\alpha +d_{0}\right)\left(\tilde{r_{p}}-1\right). \end{array}$$

When $\tilde{r_{p}}\geq 1$, *λ*_1_*λ*_2_ > 0. It is determined that all the characteristic roots of Eq. (B1) exhibit negative real parts.Consequently, the positive equilibrium point ${\tilde{E}}_{0}^{*}$ is locally asymptotically stable.

Next, we consider the global asymptotic stability of model (7) at ${\tilde{E}}_{0}^{*}$. Let

$$\begin{array}{ll} & f_{1}\left(J,N_{V}\right)=\sigma N_{V}-\alpha J-\left(d_{0}+d_{1}J\right)J,\\ & f_{2}\left(J,N_{V}\right)=\alpha J-\mu_{1}N_{V}, \end{array}$$

then

$$\begin{array}{ll} & \partial_{J}f_{1}\left(J,N_{V}\right)=-\alpha -d_{0}-2d_{1}J,\\ & \partial_{N_{V}}f_{2}\left(J,N_{V}\right)=-\mu_{1}, \end{array}$$

From this, we have $\partial_{J}f_{1}\left(J,N_{V}\right)+\partial_{N_{V}}f_{2}\left(J,N_{V}\right)=-\alpha -d_{0}-2d_{1}J-\mu_{1}<0$.

By the Bendixson-Dulac criterion, model (7) does not have a closed orbit in the positive quadrant of the *J* − *N*_*V*_ plane. Thus the positive equilibrium point ${\tilde{E}}_{0}^{*}$ is globally asymptotically stable.
